# Hijacking homeostasis: Regulation of the tumor microenvironment by apoptosis

**DOI:** 10.1111/imr.13259

**Published:** 2023-08-08

**Authors:** Christopher D. Gregory

**Affiliations:** ^1^ Centre for Inflammation Research Institute for Regeneration and Repair, University of Edinburgh, Edinburgh BioQuarter Edinburgh UK

**Keywords:** apoptosis, cancer, efferocytosis, extracellular vesicle, homeostasis, macrophage, tumor microenvironment

## Abstract

Cancers are genetically driven, rogue tissues which generate dysfunctional, obdurate organs by hijacking normal, homeostatic programs. Apoptosis is an evolutionarily conserved regulated cell death program and a profoundly important homeostatic mechanism that is common (alongside tumor cell proliferation) in actively growing cancers, as well as in tumors responding to cytotoxic anti‐cancer therapies. Although well known for its cell‐autonomous tumor‐suppressive qualities, apoptosis harbors pro‐oncogenic properties which are deployed through non‐cell‐autonomous mechanisms and which generally remain poorly defined. Here, the roles of apoptosis in tumor biology are reviewed, with particular focus on the secreted and fragmentation products of apoptotic tumor cells and their effects on tumor‐associated macrophages, key supportive cells in the aberrant homeostasis of the tumor microenvironment. Historical aspects of cell loss in tumor growth kinetics are considered and the impact (and potential impact) on tumor growth of apoptotic‐cell clearance (efferocytosis) as well as released soluble and extracellular vesicle‐associated factors are discussed from the perspectives of inflammation, tissue repair, and regeneration programs. An “apoptosis‐centric” view is proposed in which dying tumor cells provide an important platform for intricate intercellular communication networks in growing cancers. The perspective has implications for future research and for improving cancer diagnosis and therapy.

## 1 INTRODUCTION

The World Health Organization's latest statistics (to 2019 at the time of writing) indicate that cancer continues to feature in the top 10 causes of death worldwide and is especially prominent in high‐income countries.^1^ There is an ongoing need for improvements in anti‐cancer drug development based on advancement in understanding of cancer biology since, as people live longer, increasing numbers develop malignant disease and succumb to its deleterious effects. Encompassing more than 100 diseases, cancers are genetically driven rogue tissues that hijack normal physiological processes in order to expand to form aberrant organs^2^ and invade their hosts. Indeed, they form an integral, selfish, and highly adaptable part of their hosts. Transformed tumor cells carrying driving oncogenic mutations both affect, and are reliant upon, a complex support network of “normal” cells, vessels, nerves, tissue matrix components, and extracellular signaling factors—the tumor microenvironment (TME)—for their relentless progression. Their unfettered, Darwinian evolution enables cancerous tissues to appropriate, for their own ends, normal physiological regulatory measures, the very same control systems responsible for homeostasis, ultimately with deadly consequences.

Fundamental to the growth in cell numbers that lies at the heart of malignant diseases is a consistent imbalance in the cell gain/cell loss equation that favors population growth; conversely, within that simple framework, anti‐cancer therapy ambitions are designed to favor net loss of cells. One of the most important mechanisms by which cell loss is achieved in both normal and malignant tissues is through the apoptosis program that utilizes caspases, a family of cysteine‐dependent, aspartate‐directed proteases, to initiate and execute cell death. Apoptosis, an elemental homeostatic program, is important in the control of cancer not only by way of its well‐established cell‐autonomous tumor suppressive activities, but also because of its diametrically opposite, non‐cell‐autonomous pro‐oncogenic properties. The purpose of this review is to explore in depth the yin and yang of apoptosis in malignant disease pathogenesis and, in so doing, bring to the fore critical mechanisms that provide meaning to the effects of apoptosis in the TME. In particular, the highly communicative properties of apoptotic tumor cells are highlighted, from intercellular contact and secretory activities including the production of extracellular vesicles (EVs), to activation of tumor‐associated macrophages (TAMs), and orchestration of the resolution of inflammation, all of which have fundamental implications for cancer biology.

## 2 THE APOPTOSIS PARADOX IN CANCER BIOLOGY

### 2.1 Apoptosis: A fundamental homeostatic mechanism

**FIGURE 1:**
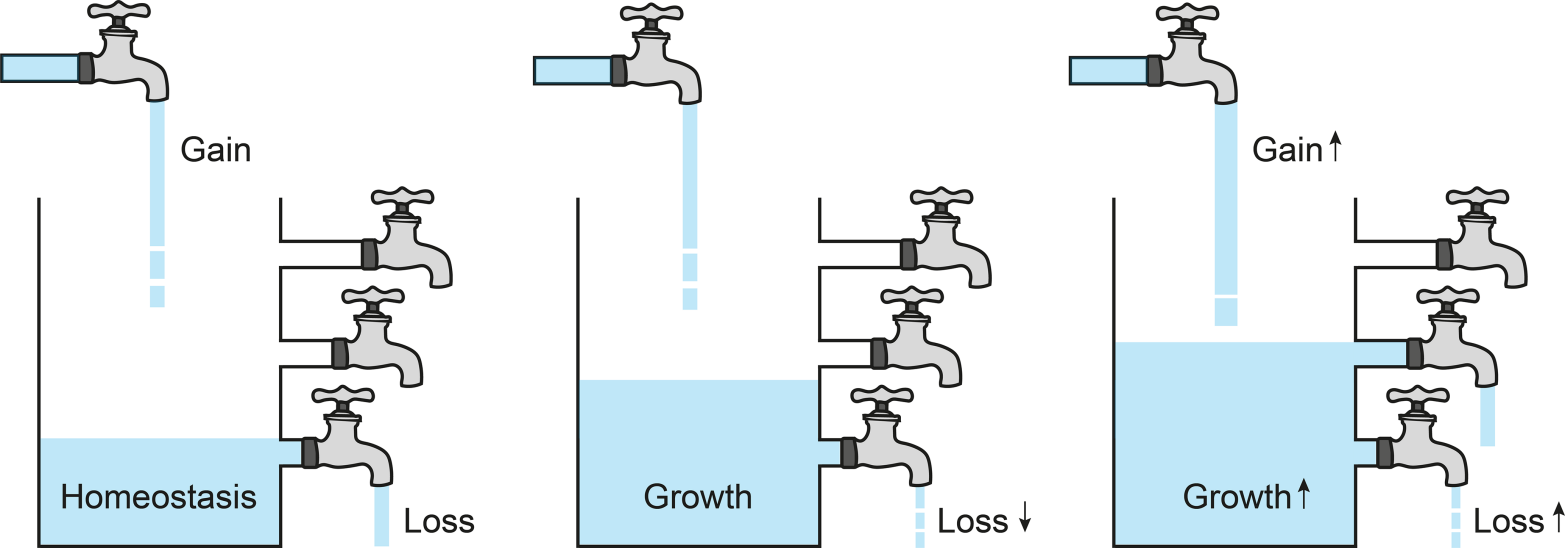
Interrelationships between cell gain, cell loss, and population growth in tumors. Input (gain) represents mitosis and inward cell migration, while output (loss) represents cell death and outward cell migration. At normal homeostasis (left tank), the rates of cell gain and loss are equivalent. Tumor growth through aberrant homeostasis may be achieved by reduction of cell loss (center tank) and/or through increase of cell gain. Variation in growth rates may occur through changes in gain and/or loss rates during the course of tumor evolution (right tank, showing increased growth). Adapted from.^4^

Homeostasis, the term originally coined by Walter B. Cannon (1871–1945) and built on the concept proposed by Claude Bernard (1813–1878),^3^ describes the self‐regulation in biological systems through which organisms' internal environments remain essentially stable in the face of external challenges. Thus, in simple terms of the self‐regulation of cell population sizes in tissues, at homeostasis the number of cells gained through cell division and/or inward migration is equivalent to the number of cells lost, through cell death and/or outward migration (Figure 1). Resetting this equation is required to increase or decrease cells as occurs during embryonic development in sculpting organs and tissues or to regulate the supply of cells needed, for example, to respond to a wound, to remove an infection or to provide a temporary function such as the lactating mammary gland. In all of these processes, the capacity to control physiologically both the production of cells by mitosis and their deletion through genetically regulated mechanisms of cell death are profound. Apoptosis the most renowned and arguably the most common of the regulated cell‐death programs (which also include pyroptosis, necroptosis, ferroptosis, entotic cell death, and immunogenic cell death among others^5^) is a key homeostatic mechanism, both in its ability to control cell numbers via direct deletion through cell suicide, but also to influence cell, tissue, and organismal homeostasis through its effects on other cells, not least those involved in the final phase of the apoptosis program, the phagocytic clearance of the cell corpses, a process known as “efferocytosis”.^6^ (The term is derived from the latin verb *efferre* meaning, in this context, “to bear to the grave, to bury” [https://en.wiktionary.org/wiki/effero]).

**FIGURE 2:**
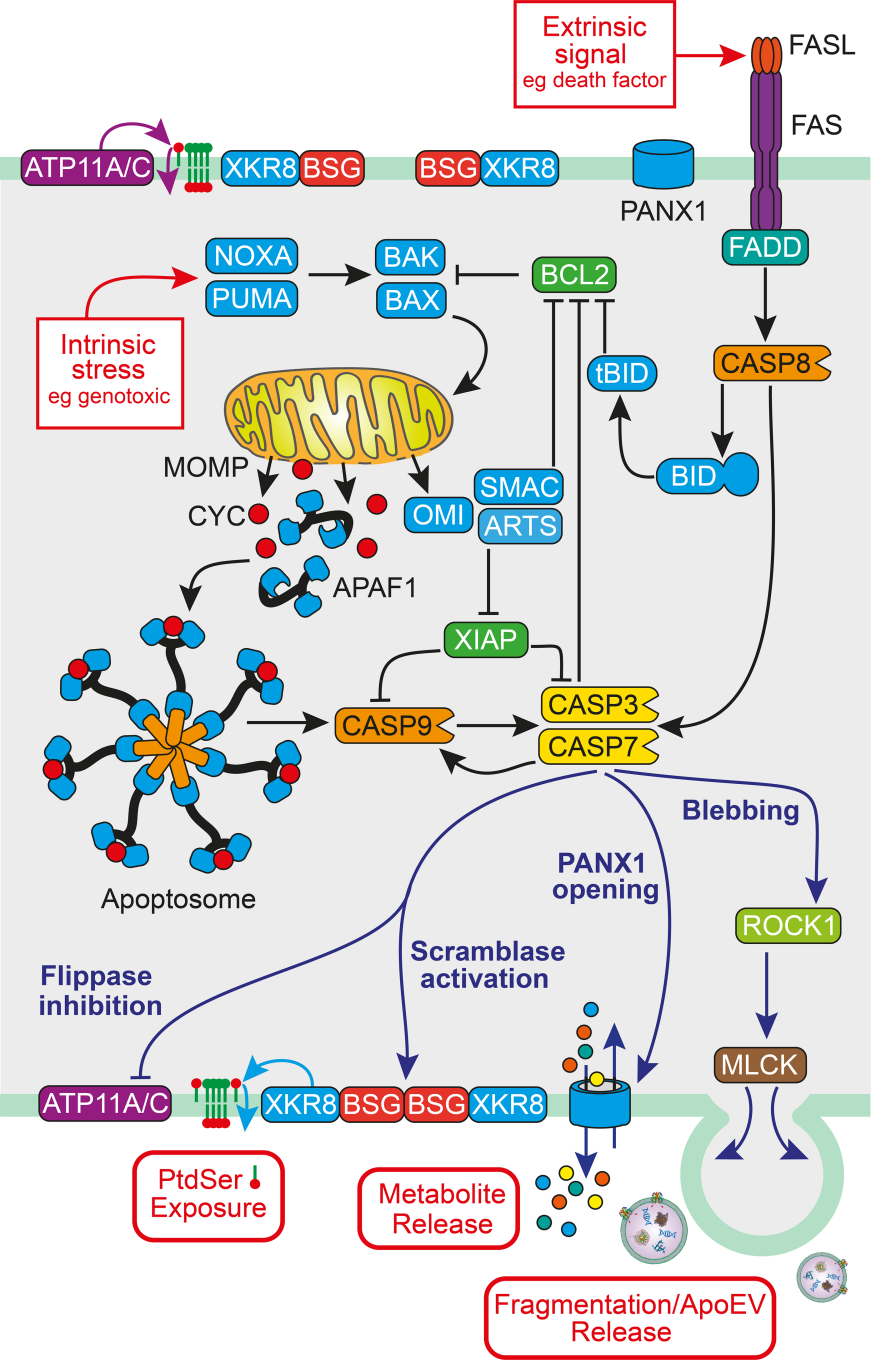
Overview of molecular pathways in apoptosis. Apoptosis is mediated by a series of caspases, cysteine proteases that only cleave after aspartate residues in the P1 position. Caspases, like apoptosis itself, are evolutionarily conserved, from worms to mammals. They are produced as zymogens and are themselves activated through proteolytic cleavage to form tetramers consisting of two large and two small fragments. In mammals, two pathways of caspase activation have been well defined: (Right) the extrinsic, or death receptor pathway and (Left) the intrinsic, or mitochondrial pathway. Prototypically, the extrinsic pathway is initiated in the plasma membrane by the clustering of transmembrane death receptors FAS, TNFR1, and DR4/5 following binding of their specific extracellular ligands, FASL, TNF‐⍺, and TRAIL, respectively. The death receptors consequently form death‐inducing signaling complexes which cleave initiator caspases 8 and 10 which, once activated, then cleave and activate executioner caspases 3 and 7. Crosstalk with the intrinsic pathway also serves to activate and amplify extrinsic apoptotic signaling. In the intrinsic pathway, multiple signals of cell stress and toxicity ranging from growth factor or nutrient deprivation to genotoxic damage, are able to initiate apoptosis by triggering mitochondrial outer membrane permeabilization (MOMP), which is controlled by the BCL2 family of apoptosis inducers and inhibitors. Among the inducers, the BH3 (BCL2 homology domain 3)‐only proteins such as tBID, PUMA, and NOXA act on the mitochondrial pore‐forming members BAX and BAK to stimulate MOMP. This allows the release of proteins, importantly cytochrome c (CYC), from the mitochondrial intermembrane space into the cytosol. Pore formation can be inhibited by the anti‐apoptotic BCL2‐family members BCL2 itself, BCL‐xL, BCLW, A1/BFL1, and MCL1. Once in the cytosol, CYC can interact with APAF1 to form the apoptosome, a heptameric cytosolic “scaffold” for the activation of caspase 9, the initiator caspase of the intrinsic pathway which goes on to activate the effector caspases 3 and 7. Additional regulation is provided by inhibitors of apoptosis (IAPs) such as XIAP and their antagonists OMI, SMAC, and ARTS. Key consequences of effector caspase activation include (lower left) rapid and sustained externalization of PtdSer through inhibition of the flippases ATP11A and C, together with activation of the phospholipid scramblase Xkr8‐BSG/NPTN; (lower center) opening of PANX1 channels enabling release of biologically active small molecules from the apoptotic cell; (lower right) induction of blebbing via MLCK phosphorylation initiated by effector caspase‐mediated activation of ROCK1, resulting in cell fragmentation/ApoEV production. (Additional outcomes of effector caspase activation of key relevance to the present perspective such as DNA cleavage and PGE_2_ synthesis are not shown).

Among regulated cell‐death programs, the defining feature of the apoptosis machinery is the cascade of caspases that both initiate and execute the internal processes that result, essentially, in the controlled demolition of the cell.^7^ A comprehensive discourse on the molecular cell biology of the apoptosis program is beyond the scope of this review and the reader is referred to excellent literature which has reviewed the molecular mechanisms of apoptosis in detail over recent years.^8^, ^9^, ^10^, ^11^, ^12^, ^13^ A brief overview is provided in Figure 2. Of note, amplification of the caspase cascade occurs via a positive feedback loop whereby effector caspase activation further activates upstream caspases. For example, a feedback amplification loop between caspase 3 and caspase 9 appears to be required for full activation of caspase 3^14^ and this is required for the acquisition of certain features of apoptotic cells such as the capacity to be rapidly efferocytosed.^15^

Thus, while their initiator caspases differ, both the extrinsic and intrinsic pathways lead to the activation of the executioner caspases 3 and 7, the latter cleaving more than 1300 substrates during the course of cellular demolition.^8^ Of particular relevance to the perspective of this review and discussed in further detail later, caspase 3 cleavages not only cause direct destructive changes in their substrates which include structural proteins of the cytoskeleton and nuclear lamins, they also generate new activities, for example: (a) the caspase‐activated DNAse responsible for the internucleosomal DNA cleavage that characterizes apoptosis, (b) activation of the phospholipid scramblase Xkr8‐BSG/NPTN that drives changes in phospholipid topology of the plasma membrane of apoptotic cells, (c) induction of blebbing and fragmentation of the cell through activation of the Rho‐associated coiled‐coil‐containing protein kinase 1 (ROCK1) and (d) secretory activity, including the opening of Pannexin 1 (PANX1) channels in the plasma membrane that enable the release of biologically active small molecules from the apoptotic cell (Figure 2). These represent critical aspects of the apoptosis program, from the demolition of the cell to its capacity to undertake intercellular communication and to signal its clearance. Therefore, the proximal signaling routes to the extrinsic and intrinsic apoptosis pathways, the expression of pro‐ and anti‐apoptotic BCL2 family members, among other apoptosis regulatory proteins such as inhibitors of apoptosis (IAPs) and their antagonists, and the activation of caspases are all crucial control points in a cell's apoptosis decision‐making and as such constitute important cellular homeostatic elements of the apoptosis program of relevance to the dynamics of cell gain and loss in the TME.

### 2.2 A brief history of cell death in cancer—A pathological perspective

In 1967, the Norwegian pathologist Olav Iversen aptly stated: “Normal renewing tissues have a cell loss which is 100% of the cell renewal. Tumours have a reduced cell loss”.^16^ This fact is appropriately captured in Hanahan and Weinberg's landmark perspectives of the hallmarks of cancer in which they list “evading apoptosis” (later “resisting cell death”) as one of the key acquired capabilities of most, perhaps all, cancers.^17^, ^18^ This may however understate the levels of cell loss and cell death that are observed in tumors. For example, in several types of aggressive cancers that have been studied in terms of their actual growth in relation to their cell doubling time (DT) it has been found that cell loss is substantial. While the actual DT of cancers of particular types and grades various substantially, calculation of the cell loss factor (CLF) as originally proposed by Steel^4^, ^19^ produces useful data on the extent of cell loss in relation to cell gain in malignant diseases. CLF relates the actual DT of a tumor to its potential DT_pot_, the latter being the calculated DT of the cancer cells themselves derived historically by tritiated thymidine labeling studies. CLF is described as 1 − DT_pot_/DT and therefore the fraction of cells produced by mitosis which are lost. For normal tissue homeostasis, the steady state would predict cell gain to equal cell loss, as Iversen pointed out, producing a CLF of 1 (or 100%). However, with no cell loss (DT equal to DT_pot_) CLF would be zero and so if growth of tumors were due in most part to proliferation, CLFs would approach zero. This is by no means the case; rather, CLFs in malignancies of various histological types and grades tend to be greater than 50%, often much greater and may exceed 90% (90% of the cells gained by mitosis being lost). For example, an investigation of a range of human lung cancers determined CLFs of >70% for all primary lung neoplasms, with the majority of primary bronchogenic and all undifferentiated and large cell carcinomas showing ≥90% losses of the cells gained.^20^ Adenocarcinomas have been reported to lose >70% of cells gained, while squamous cell carcinomas, lymphomas, and embryonal tumors lose >90%.^21^ Rectal adenocarcinomas^22^ and primary bronchogenic carcinomas^20^ may lose as much as 98% of the cells they produce. Other examples of substantial cell loss in both human and experimental animal tumors have been reported.^23^ In general, in malignant diseases, as is the case for cell turnover in healthy tissues, cell loss increases when cell proliferation rises.^4^, ^20^ This may be due to inherent mechanisms that guard against the dangers of cell division, such as apoptosis of cells carrying DNA damage and/or as a consequence of other apoptosis‐inducing stresses like limitation of nutrients, oxygen, and growth factors. It is therefore interesting to note Iverson's controversial proposal of over half a century ago that, while cancer was classically considered as a more rapidly proliferating tissue than its normal counterpart, an alternative view would be that of a renewing tissue with a marginally reduced cell loss.^16^

Cell loss in tumors is commonly due to cell death (although other mechanisms such as differentiation and migration also contribute) and, as they increase in size, malignant tumors frequently display significant regions of histologically clear necrosis—accidental cell death of large numbers of cells—occurring as a consequence of tumor proliferation outpacing essential oxygen and nutrient supplies (conditions which also trigger apoptosis). Such increases in cell death as tumor growth proceeds ultimately cause growth decline.^24^ Apoptosis too is common in malignant diseases; seminal definitions of apoptosis were made in the context of cancer pathology as well as in normal tissue turnover and embryonic development. Writing in 1972 in the British Journal of Cancer, Kerr, Wyllie, and Currie proposed the term “apoptosis” (from the Greek meaning “falling of leaves or petals”) to describe a type of cell death that differed markedly from necrosis: In contrast to necrotic lesions that signify large tracts of dead cells whose morphology is poorly preserved, apoptosis was clearly discernible in discrete cells in scattered locations. Characterized mainly by transmission electron microscopy, apoptotic cells were recognized by their shrunken size, condensed chromatin, often well‐preserved cytoplasmic organelles and nuclear fragmentation, markedly contrasting with necrotic cells which appear swollen, with poorly preserved organellar structure. Fragmentation of the cells into apoptotic bodies was often observed, as was their engulfment and lysosomal degradation by neighboring cells in the absence of any evidence of inflammation.^25^, ^26^ Kerr and colleagues proposed that apoptosis is a regulated mode of cell death which, because of its occurrence without tissue‐damaging side effects, would be “well suited” as a mechanism of tissue homeostasis.^25^ In the context of cancer they noted, “Both apoptotic bodies and mitotic figures are sometimes numerous in rapidly growing tumours; it is the balance between the two processes that determines the rate of enlargement”.^25^ These fundamental imbalances between proliferation and apoptosis that provide a simple platform upon which the additional complexities of cancer can be built have been eloquently discussed elsewhere.^17^, ^18^, ^27^

The relatively high frequency with which apoptosis can be recognized histologically in many neoplastic tissues (although in absolute terms numbers of discernible apoptotic cells are usually small) is suggestive of high or very high rates of cell death via the apoptosis program. This is because the efficiency of efferocytosis and subsequent degradation makes the window of opportunity for recognizing an individual apoptotic cell all too brief, estimated at around 1–3 h.^28^ Indeed, the swift corpse clearance or rapid loss from surfaces into lumina, blood, or external environment has made detailed assessment of the rate of apoptosis in all tissues, both healthy and neoplastic, difficult and very likely underestimated. Inhibition of engulfment can help reveal more accurately the levels of apoptosis in tissues,^29^ but new in situ technologies which permit monitoring of the apoptosis program from the suicidal commitment of the cell right through to its engulfment by phagocytes (see, e.g.,^30^) will undoubtedly revolutionize our understanding of the role of apoptosis in tissue kinetics and help further clarify its roles in cancer. The short‐lived display of apoptosis in situ has parallels with mitosis; mitotic figures are also only fleetingly visible in standard tissue sections because of the brevity of the histologically recognizable mitotic phases. Therefore, the appearance of numerous apoptotic cells and bodies, along with abundant mitotic figures in typical histological snapshots, is highly indicative of both rapid proliferation and substantial cell death.

Realization of the significance of apoptosis in determining cell population growth and shrinkage rates, the latter illustrating its potential as a therapeutic target, motivated researchers both to assess the rates of apoptosis in a spectrum of malignant diseases and to determine whether apoptosis rates could be accelerated in response to anti‐cancer drugs and radiation. Results of these studies have tended to be morphometric rather than detailed tissue kinetics and a selection are presented in Table 1. Various approaches have been taken to measure apoptosis in histological sections of malignant tumors, ranging from standard hematoxylin and eosin morphological criteria to DNA fragmentation (in situ end labeling) and effector caspase activation assessment. A common variable is the “Apoptosis Index” (AI) which relates the number or area of apoptotic events recorded per unit area of tumor section to the total number/area of cells observed. Given the difficulties in quantifying apoptosis accurately in tissue sections, AI measurements seem likely to underestimate true apoptosis levels. Notwithstanding these limitations, relatively high rates of apoptosis as determined by AI in a range of fully established malignant diseases often correlate directly with disease aggressiveness (Table 1). Although this relationship does not by any means prove causality, it is noteworthy that, in multivariate analyses, AI can be the only prognostic indicator of overall survival (pancreatic ductal adenocarcinoma,^32^) and as an indicator of disease aggressiveness is sometimes also a predictor of disease recurrence (colorectal carcinoma,^43^). Further work will be required to establish a causative role for apoptosis—rather than representing simply the known close‐coupling of proliferation and apoptosis^45^—in different types of cancers.

**TABLE 1:** Illustrative examples of apoptosis indices (AI) in relation to prognosis in various cancers (non‐exhaustive list).

Cancer type	Classifications	Measurement of apoptosis	Findings	Reference
Pancreatic carcinoma	Ductal adenocarcinoma, Stages I–III	In situ end labeling (ISEL, (histology)	Spontaneous apoptosis associated with poor prognosis	[31]
Pancreatic carcinoma	Ductal adenocarcinoma	ISEL (histology)	Higher AI is prognostic (shorter median survival). In multivariate analysis, AI was the only prognostic indicator of overall survival	[32]
Breast carcinoma	Primary and lymph node metastasis	ISEL (histology)	High AI correlates with multiple poor prognostic factors: poorly differentiated carcinomas, large tumor size, high proliferative and mitotic indices. Higher AI in lymph node metastases than primary tumors	[33]
Glioblastoma (GBM, highest grade astrocytoma)	With pseudopalisades (necrotic cores with hypercellularity at periphery. These are pathognomonic of GBM)	Active caspase 3 (compared with adjacent astrocytoma)	More apoptosis and less proliferation. Hypercellularity in these regions due to migration of cells from occluded blood supply	[34]
Astrocytic tumors	GBM, diffuse astrocytoma, anaplastic astrocytoma	ISEL (histology)	AI strongly positively correlated with proliferation index in GBM but only in p53‐negative tumors	[35]
Brain metastases	Lung, breast, kidney, melanoma, and unknown primaries	Anti‐SS DNA	AI strongly positively correlated with proliferation index	[36]
Non‐small cell lung cancer	Squamous and adenocarcinoma	ISEL (histology)	High AI and proliferative index in squamous	[37]
Gastric carcinoma	Differentiated and undifferentiated types	Morphology in H&E, AI and mitotic index	AI lower in early as compared to advanced stage disease, positively correlated with mitotic index	[38]
Colorectal carcinoma	Dukes stage C and D with liver and/or lymph node metastases	ISEL (histology)	Both proliferation and apoptosis indices increased in metastatic foci	[39]
Colorectal carcinoma	Primary and metastases	ISEL (histology)	AI prognostic indicator: higher AI = poorer prognosis	[40]
Colon and rectal cancers	Dukes A–D	ISEL (histology)	High AI correlated with poor prognosis in colon cancer	[41]
Colorectal carcinoma	Dukes A–C	M30 (caspase cleaved cytokeratin 18 fragment)	High AI correlated with local recurrence post‐surgery and metastasis	[42]
Colorectal carcinoma	WHO I–IV	ISEL (histology)	High AI prognostic of poor overall survival and poor disease‐free survival. Increased recurrence rate with high AI	[43]
Cholangiocellular Carcinoma	CCC, TNM stages I–IV; other clinicopathological features	AI by morphology in H&E	Higher AI and lower BCL2 expression associated with more aggressive disease: higher Ki67 labeling, lymph node metastasis, and vascular invasion	[44]

### 2.3 Tumor‐suppressive and tumor‐progressive roles of apoptosis: Unifying the paradox

The tumor‐suppressive properties of apoptosis are intuitively sensible, well accepted, especially for BAX/BAK‐dependent apoptosis, and well‐reviewed elsewhere.^11^, ^13^, ^17^, ^18^, ^46^ Most of the regulated cell death of mammalian tissue turnover is likely to occur through the mitochondrial pathway of intrinsic apoptosis in response to multiple stresses. As already discussed, this intrinsic pathway is finely tuned and controlled by the BCL2 protein family. Available evidence supports the view that inappropriate cell survival from the onset of the oncogenic process, for example via failure of p53 to initiate apoptosis (usually because of mutations that are common in various cancers) to signal apoptosis by transcriptional activation of the pro‐apoptotic BH3‐only BCL2‐family members PUMA and NOXA, is likely to be a key pathway in multistep oncogenesis enabling survival of cells harboring harmful DNA damage.^47^ BCL2 itself, the prototypic oncogene that operates by promoting cell survival,^48^ can prevent apoptosis, along with other anti‐apoptotic members of its family, in response to numerous stresses to which pre‐malignant or rapidly expanding malignant cells are exposed. These include genotoxic damage, oxidative and metabolic stresses, and limited growth factor availability. Unsurprisingly, anti‐apoptotic BCL2 family proteins are commonly reported as being upregulated in aggressive cancers while pro‐apoptotic members are often down‐regulated (reviewed recently in^11^). In the classic case of the apoptosis‐suppressing oncogene BCL2, which was originally identified in the t(14;18) chromosomal translocation in follicular lymphoma,^49^ the resultant de‐regulated suppression of apoptosis in B cells initially causes only an indolent, slowly proliferating and slow‐growing disease; this may evolve into an aggressive malignant disease once additional mutations have been accrued.^50^, ^51^ Another important example of a regulated survival pathway that is activated in many cancers involves AKT/PKB which, as part of the PI(3)kinase pathway, helps couple receptor tyrosine kinase signaling to the apoptosis regulatory circuitry. In lymphomagenesis, AKT was originally found to activate the serine/threonine kinase, mTOR, a master regulator of cellular homeostasis^52^ in this case operating upstream of the translational controller, eIF4E.^53^

Suppression of apoptosis appropriately coupled with ongoing or accelerated proliferation is likely to form a platform for further oncogenic evolution, as already noted. The levels of each process are likely to characterize different tumor types and grades. As well as cell loss and relatively high levels of apoptosis being observed in aggressive, rapidly growing cancers it is also noteworthy that slow‐growing, highly proliferative tumors may occur in which the number of tumor cells gained by mitosis is almost completely balanced by the apoptotic cell number. This is the case with basal cell carcinoma, a common, locally invasive malignancy that grows very slowly as a consequence of its high rate of apoptosis.^54^, ^55^ A similar principle features in metastatic growth control. Thus, dormancy of lung metastases in mice can be achieved by suppression of angiogenesis which triggers increased apoptosis. This balances proliferation and effectively prevents metastatic growth; when angiogenesis occurs, apoptosis is reduced without change in proliferation rate and metastatic growth proceeds,^56^ thus proving experimentally Iversen's prediction.^16^

**FIGURE 3:**
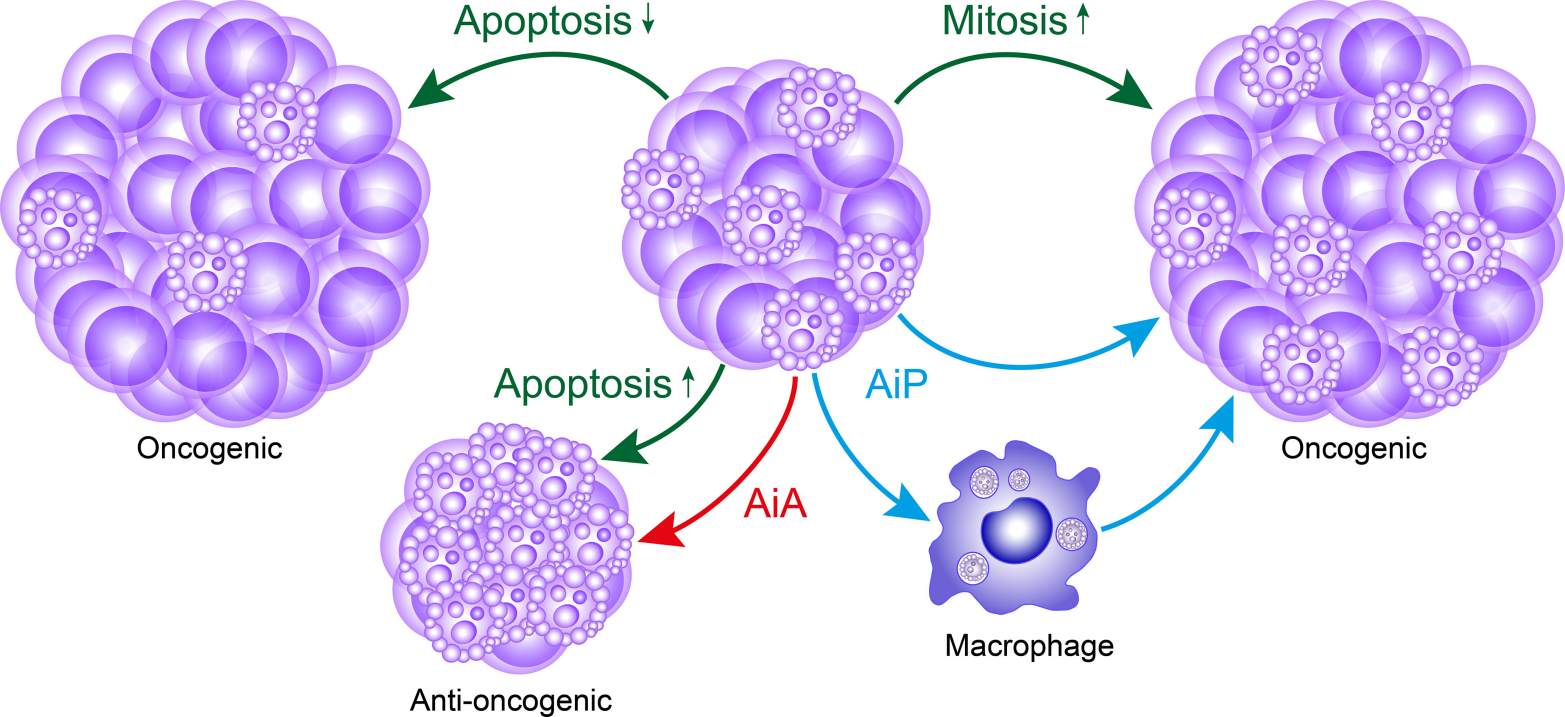
Cell‐autonomous and non‐cell‐autonomous effects of apoptosis on tumor growth. Decreased apoptosis or increased mitosis through cell‐autonomous programs (green arrows) have oncogenic implications, each leading to growth, which may also be facilitated through non‐cell‐autonomous, growth‐promoting effects of apoptosis such as AiP (apoptosis‐induced proliferation [aka compensatory proliferation], blue arrows). AiP may be induced directly by mitogens released from apoptotic cells or via interactions with other cells, notably efferocytosing tumor‐associated macrophages. Anti‐oncogenic increases in cell death (as induced, for example by anti‐cancer therapies) may also be mediated either by cell‐autonomous apoptosis or by non‐cell‐autonomous AiA (apoptosis‐induced apoptosis, red arrow).

The evidence that apoptosis can either be relatively high or low in both aggressive and benign tumors can be rationalized in simple terms by the unbalancing effects of proliferation since tumor cell population expansion obviously cannot be achieved in the absence of mitosis. This principle works cell‐autonomously: to achieve net increases or decreases in tumor‐cell population sizes needs only mitotic or apoptotic activities, respectively, of individual tumor cells (Figure 3). It is obvious that apoptosis of a single, pre‐malignant mutant cell deletes that cell and therefore theoretically provides an effective surveillance mechanism that militates against cancer emergence from the outset. This cell‐autonomous scenario implies a tumor‐suppressive role only for the apoptosis program and its machinery: the apoptotic cell dies quietly and is gone. The problem with this simple viewpoint is that, although apoptosis is commonly “quiet” from the perspective of acute inflammation, the program is far from silent.^57^ Rather, the actively dying cells are powerful signaling entities that can have profound effects on their surroundings. Interrelationships between inflammation, apoptosis, and oncogenesis serve to illustrate this point. For example, in the liver, deficiency in the NF‐κB essential modulator, NEMO in liver parenchymal cells leads to spontaneous apoptosis that drives development of hepatocellular carcinoma (HCC) in mice. NEMO proved to control liver parenchymal cell survival (through both NF‐κB‐dependent and ‐independent pathways) by suppressing the formation of RIPK1/FADD/caspase 8‐signaling complexes of the extrinsic apoptosis pathway.^58^ Exactly how hepatocyte death leads to chronic liver disease and eventually HCC remains to be determined but very likely involves the dying cells acting to condition, through extracellular signaling properties, the chronic inflammatory environment which may be regarded, essentially, as a nascent TME. Here, apoptosis is shown to have oncogenic properties through its non‐cell‐autonomous effects (Figure 3) and, as we shall see, these have significant implications for emerging and established cancer tissues, as well as for tumor repopulation following cell death‐inducing anti‐cancer therapy.

## 3 PROGRAMMING OF THE TUMOR MICROENVIRONMENT BY APOPTOSIS

The concept that primary cancers and metastases are dysregulated, rogue tissues consisting of both tumor cells and non‐transformed host cells, rather than collections of tumor cells only, is rooted firstly in Rudolf Virchow's suggestion in 1863 that cancers originate at sites of chronic inflammation,^59^ and secondly in the “seed and soil” theory of metastasis, originally advanced by the English surgeon, Stephen Paget in 1889.^60^ Despite this long‐standing conceptual framework that, in order to thrive, tumor cells require a supportive milieu, the TME, the primary focus of cancer biology and medicine for the past 50+ years has largely been the transformed tumor cell and its genetic predisposition to cell‐autonomous proliferation. It has steadily become clearer over more recent years, however, that the TME is a critically supportive feature of all cancers (primary, metastatic, and relapsed) and as such provides targets for anti‐cancer therapies. Supporting the transformed tumor cells, the TME comprises resident and recruited stromal cells and various tissue matrix components. The stromal cellular elements include endothelial cells, pericytes and fibroblasts as well as immune stromal cells such as T and B lymphocytes, NK cells, macrophages and other myeloid cells, mast cells and platelets.^61^ Inflammation has long been known to promote carcinogenesis and it is now becoming clearer that principles established in mouse models, notably the promotion to malignant lesions of dormant cells carrying oncogenic mutations through stimulation of inflammation^62^ also applies in multistep carcinogenesis in humans.^63^ The histopathological picture of the TME has been likened to “wounds that do not heal”,^64^ although an improved description based on the inflammatory responses in wound healing is that of “wounds in perpetual resolution,” a state that might be expected in the context of constitutive apoptosis. Cellularity of TMEs is variable, most obviously between different cancer types but even within different regions of individual cancers. Diversity is broad and certain tumor types display dominance of specific stromal cells. For example in Hodgkin's lymphomas, many different recruited cell types abound in the TME, including multiple classes of T cells, B cells, plasma cells, eosinophils, mast cells, and macrophages, whereas the Reed‐Sternberg tumor cells constitute the minority cell in the tumor mass. By contrast, in Burkitt's lymphoma (BL), the tumor cells are the most common cell type, while the predominant stromal cellular elements of the TME are TAMs.^65^

**FIGURE 4:**
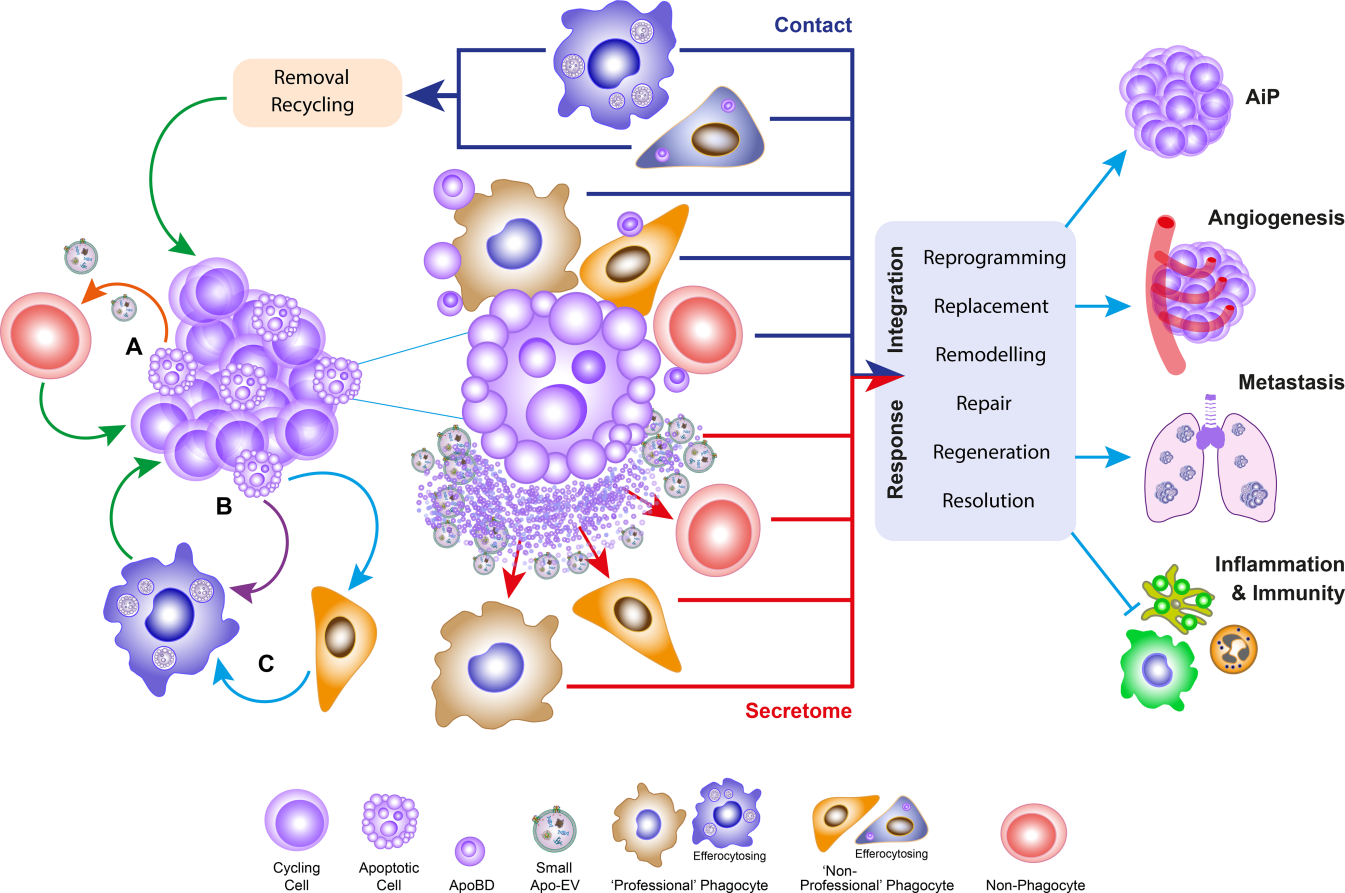
Overview of the potential oncogenic effects of apoptosis in tumor tissues. Representation of intercellular signals from apoptotic cells in a growing tumor, which are mediated either through the apoptotic secretome, including small ApoEV production (lower center, red pathways), or via contact‐dependent means (upper center, dark blue pathways). Response signals are integrated into pro‐oncogenic effector programs (right) such as trophic apoptosis‐induced proliferation (AiP), angiogenesis, invasion, enhanced migration and metastasis, resolution and anti‐inflammatory signaling as well as suppression of pro‐inflammatory effects and of innate and adaptive anti‐tumor immunity. Recycling of digested apoptotic cell‐derived components (top left) also very likely helps support tumor growth. Macrophages probably play critical roles in many of these responses and comprise the major professional efferocytic cells of the TME and probably respond, independently of efferocytosis to contact‐mediated and secretory communication from apoptotic cells too. Notably, virtually any cell in the malignant tissue has the potential to respond to the various signals emanating from apoptotic cells, some acting as professional efferocytes. In this way, it is proposed that intricate, intercellular circuits (left), founded in apoptosis, can be established to support disease development. Illustrative, theoretical examples of such circuits are shown in which the endpoint in each case is pro‐oncogenic (green arrows). In circuit A (orange arrow), contactless stimulation of a non‐phagocyte (e.g., lymphocyte) of the TME is shown. In B (purple arrow), the response is that of an efferocytosing macrophage, while C is more complex (blue arrows) depicting communication between non‐professional and professional phagocytes. See text for molecular pathways that may contribute to these, and other, circuits.

Current understanding of the TME is that tumor and stromal cell populations participate in bidirectional crosstalk through paracrine and juxtacrine signaling pathways that support key oncogenic programs such as survival, proliferation, suppression of innate and adaptive anti‐tumor immunity and promotion of angiogenesis and metastasis.^61^, ^66^, ^67^, ^68^, ^69^ Critically, the TME also shapes the metabolic profile of cancers.^70^ In contrast to their healthy forebears, apoptotic tumor cells have up to now only rarely been regarded as contributors to the supportive activities of the TME, even though their tumor‐promoting effects were noted long before the term “apoptosis” was coined.^71^ Yet the case for their inclusion is compelling for several reasons (Figure 4): (1) Apoptotic tumor cells are frequent members of the cellular community of various cancers (Table 1); (2) they are highly active intercellular communicators (see Section 3.1); (3) they exhibit well‐known immunomodulatory characteristics and drive resolution of inflammation^72^, ^73^; and (4) they can direct, as discussed below, additional pro‐oncogenic programs, such as proliferation, angiogenesis, and migration, in cells with which they communicate. As outlined in the next sections, mounting evidence indicates that apoptotic tumor cells have persuasive potential to promote oncogenic activities in the TME, not least through their capacity to activate TAMs and through the activities of their EVs and other secretory components (Figures 3 and 4). Although, in this context, the main focus of this perspective is on the pro‐oncogenic activities of apoptotic tumor cells, such properties have also been reported for other stromal cells (mouse embryonic fibroblasts, MEFs).^74^

### 3.1 Communicative properties of apoptotic cells in the TME

Far from endowing inert and effete characteristics upon cells in which it is activated, the apoptosis program engenders potent long‐ and short‐distance signaling properties, which have important implications for tissue homeostasis, be it normal or neoplastic. Such signals are broad‐ranging, from aiding swift removal of the dying cell to recycling its constituent parts; repair, remodeling and tissue regeneration; replacing lost cells, reprogramming neighbors and resolving inflammatory events (Figure 4). Although context‐dependent details remain largely undiscovered, much of our present knowledge of the capacity of apoptotic cells to act as signaling beacons is based on (i) the plasma membrane changes that mark the cell's altered relationship with its microenvironment and that presage (ii) organized cellular dismantling and fragmentation (generation of apoptotic bodies, ApoBDs), (iii) activation of secretory pathways, (iv) binding and uptake of apoptotic cells by phagocytes (efferocytosis), and (v) modulation of phagocyte programs through interaction with apoptotic cells and their products. These elements of the apoptotic cell's capacity to communicate with its tissue environment provide a useful categorization of the apoptosis‐induced mechanisms at play in the TME, not only from the perspective of efferocytosis in tumors but also in determining the wide variety of cancerous tissue responses to apoptosis of its constituent tumor cells and stromal cells.

#### 3.1.1 Plasma membrane changes induced by apoptosis

Profound plasma membrane changes that are induced by the apoptosis program lie at the root of the apoptotic cell's ability to alter the ways it interacts with its environment. Gross structural changes such as cell shrinkage, loss of microvilli, and surface blebbing of the cell^25^, ^75^ are accompanied by radical changes in plasma membrane structure and function. These changes involve lipids, proteins, and carbohydrates that not only mediate alterations in the interactive capacity of the plasma membrane but also control the permeability of the dying cell along with its capabilities to secrete and to fragment.

Elemental changes in phospholipid topology are among the best understood molecular processes that occur as a consequence of apoptosis. It has been known for over three decades that the aminophospholipid, phosphatidylserine (PtdSer), which is actively translocated to the inner leaflet of the plasma membrane in healthy cells, becomes externalized in apoptotic cells prior to loss of membrane integrity and serves as an “eat‐me” signal for phagocytes.^76^ Phosphatidylethanolamine (PtdEtn), the other main aminophospholipid of the plasma membrane, is translocated similarly to reside on its inner facing leaflet in healthy cells.^77^ It has become clear that the ATP‐dependent aminophospholipid translocases, ATP11A and ATP11C, which actively flip PtdSer (and PtdEtn) to the inner plasma membrane leaflet in healthy cells, are irreversibly inhibited during apoptosis through effector caspase cleavage (reviewed recently in^78^). Rapid externalization of PtdSer in apoptotic cells is achieved through the action of the ubiquitously expressed XK‐related protein, Xkr8, which resides in the plasma membrane in healthy cells as a heterodimer complexed with one or other of the immunoglobulin superfamily proteins, Basigin (BSG) or Neuroplastin (NPTN). During apoptosis, caspase‐mediated cleavage of the C‐terminal domain of Xkr8 irreversibly activates the Xkr8‐BSG/NPTN complexes to form heterotetramers which have phospholipid scramblase activity. In this way, PtdSer is rapidly externalized and, because flippase activity is simultaneously crippled, remains exposed on the external leaflet of the apoptotic cell's plasma membrane.^78^, ^79^, ^80^ This mode of PtdSer exposure, which is required for phagocytosis of apoptotic cells, is conserved from nematode worms to mammals, the *Caenorhabditis elegans* Xkr8 orthologue being CED‐8.^79^

While PtdSer exposure on apoptotic cells appears to be a necessary pre‐requisite for efferocytosis—both by macrophages and by other, non‐professional phagocytes such as fibroblasts^81^—it is not sufficient. Much evidence supports this viewpoint (see for example^82^), perhaps the most compelling being the failure of macrophages to engulf viable cells that expose PtdSer driven by constitutive activation of the Ca^2+^‐dependent phospholipid scramblase TMEM16F.^83^ Many additional apoptosis‐induced plasma membrane changes have been observed which may contribute to the binding and uptake of apoptotic cells by phagocytes as well as potentially other signaling properties too. Examples include alterations in sugar exposure such as clustering of sialylpolylactosaminyl chains of CD43,^84^ qualitative and quantitative changes in proteins such as Annexin A1 and ICAM‐3 (the former translocating from the cytosol to the apoptotic‐cell surface,^85^ the latter undergoing conformational change on apoptotic cells^86^), binding of oxidized low density lipoprotein,^87^ colocalization of calreticulin (CRT, translocated to the surface from the ER) with exposed PtdSer,^88^, ^89^ and the appearance of binding sites for proteins such as complement components, thrombospondin 1 (THSP1), and IgM, as well as cross‐reactive epitopes for anti‐LPS antibodies.^90^, ^91^, ^92^, ^93^, ^94^ Thus, qualitative and topological changes in membrane components—including redistribution of membrane proteins and lipids, together with modifications in their structures for example through oxidation or glycosylation—in addition to quantitative changes in surface expression are key principles that underpin the functional attributes of the apoptotic cell plasma membrane.

Importantly, healthy cells are protected from phagocytosis by surface expression of “do not‐eat‐me” signals (reviewed recently^95^). The best characterized of these is CD47, an ubiquitously expressed immunoglobulin superfamily member and “self” determinant, which engages with SIRP⍺ on phagocytes, activating inhibitory signaling via its intracellular ITIM motifs via the SH2 domain‐containing phosphatases SHP‐1 and SHP‐2, and thus provides an effective means to prevent phagocytes mounting responses to healthy cells. This “do not‐eat‐me” signaling axis is suppressed in apoptosis either through loss of CD47 from the apoptotic cell surface or by disruption of CD47's capacity to cluster in association with lipid rafts.^15^, ^88^, ^96^ Paradoxically, CD47 also seems able to act as a tethering receptor in efferocytosis^97^; this is potentially due to apoptosis‐induced qualitative changes in its structure or topology that may include acquisition of capacity to bind THSP1.^95^ Antibody blockade of CD47 provides an effective means of inducing phagocytosis and degradation of viable cells, and this form of antibody‐dependent phagocytosis is showing much promise in treating a spectrum of cancers.^98^ A further “do not‐eat‐me” signal, of potential importance in clearance of apoptotic leukocytes, involves homotypic CD31‐CD31 interactions in trans, which appear to be important for the prevention of avid intercellular adhesion of healthy cells under conditions of low‐shear flow. Through mechanisms which remain unclear, CD31 of apoptotic cells acquires the ability to tether strongly to macrophage CD31 in order to facilitate efferocytosis under flow.^99^ In addition to SIRP⍺, a spectrum of immunoglobulin superfamily anti‐phagocytic receptors that also operate via ITIM motifs have been identified in mice and humans. These include CD22 (SIGLEC‐2), CD33 (SIGLEC‐3), CD300a, CD300f, PD‐1, SIGLEC‐10 (human), SIGLEC‐G (mouse), and LILRB1 (human). Their roles in regulating efferocytosis remain either unexplored or, as yet, unclear (reviewed in^95^).

Together, these fundamental changes in plasma membrane architecture provide apoptotic cells with new opportunities for intercellular communication, notably with macrophages and other efferocytes, via a spectrum of phagocyte receptors capable of generating signals that trigger engulfment and also modulate inflammation and immunity.^100^ Exposure of PtdSer permits interactions with a variety of receptors which either link directly with the apoptotic cell‐borne PtdSer or indirectly through coupling to PtdSer‐binding intermediaries. Direct PtdSer receptors include the pattern recognition receptor BAI1,^101^ Stabilin‐2,^102^ TIM4,^103^ TLT2,^104^ and RAGE.^105^ Indirect PtdSer interactions involve (1) members of the TYRO3‐AXL‐MER family of receptor tyrosine kinases, notably MER, which signals anti‐inflammatory responses to apoptotic cells via coupling proteins GAS6 or PROS1 which have PtdSer‐binding abilities in their amino‐terminal GLA domains^106^; (2) MFG‐E8 (lactadherin) which couples exposed PtdSer to integrins ⍺_v_β_3_ and ⍺_v_β_5_ on phagocytes,^107^, ^108^ an activity that may be promoted by oxidation of PtdSer^109^; and (3) a spectrum of other opsonins including C1q and the lung surfactant proteins SP‐A and SP‐D. C1q binds to PtdSer on apoptotic cells^110^ (but note also below that C1q has lectin activity too^111^) allowing it to interact with the efferocytic receptors CD91/LRP1^112^ and SCARF1.^113^ SP‐A and SP‐D can opsonise apoptotic cells by PtdSer binding and interact with CD91/LRP1 on alveolar macrophages in complex with CRT, enabling efferocytosis^114^; SP‐A and SP‐D appear also capable of inhibiting alveolar macrophage phagocytosis by ligating SIRP⍺.^115^ Non‐PtdSer membrane changes have the potential to connect the apoptotic cell independently of PtdSer with efferocyte receptors such as scavenger receptors (e.g., CD36 (via THSP1 bridging)^91^), other pattern recognition receptors (e.g., CD14^116^), complement receptors, (e.g., CD91/LRP1 (via mannose‐binding lectin, C1q and CRT)^112^; note that C1q may also be recognized by phagocytes in the context of CRT and PtdSer^117^), integrins (e.g., ⍺_v_β_3_; via THSP1^118^) and C‐type lectin receptors such as MGL1^119^ and LSECTin.^120^ Theoretically, any of the apoptotic cell surface membrane modifications that lead to signaling in neighboring cells have the potential to modulate the TME. Therefore, many options are available, at least in theory, to support complex, context‐dependent intercellular communication pathways in response to apoptosis in cancer tissues.

Finally in this section, apoptosis‐induced changes in plasma membrane channel activities regulate bidirectional transfer of ions, water and larger bio‐active molecules between the intracellular and extracellular milieux of the dying cells. Indeed, ionic movements controlled by the Na^+^/K^+^‐ATPase may well be important in determining thresholds for apoptosis signaling.^121^ Of particular relevance to the present perspective are observations over recent years that PANX1, a broadly expressed oligomeric membrane channel with diverse known functions in health and disease (including inflammation and cancer), is directly and irreversibly activated in apoptosis to regulate transfer of biologically active, relatively large molecules (up to approximately 1 kDa) from the cytosol of the dying cell to the external microenvironment. Thus, subunit cleavage by effector caspases 3 and 7 produces an open PANX1 channel configuration that readily permits passage of ATP, UTP and other large signaling metabolites such as glutamate and spermidine.^122^, ^123^, ^124^ As we will see, PANX1 channel opening has important implications both for the secretory properties of apoptotic cells in promoting efferocytosis and tissue repair and for their controlled disassembly.

#### 3.1.2 Orderly dismantling of apoptotic cells into apoptotic bodies

A classical characteristic of apoptosis is the cell's capacity to orchestrate gross changes in its structural architecture as it shrinks and very frequently dismantles itself through production of membrane‐delimited subcellular fragments known as ApoBDs. ApoBDs may be regarded as a subtype of the EVs produced by apoptotic cells (ApoEVs) with sizes reported to range frequently from around 1 to 5 μm (although they may extend beyond either end of this range).^125^ For the purposes of the present perspective, the term ApoBD will be used to describe all relatively large ApoEVs, arbitrarily of ≥1 μm and therefore visible by light microscopy and also readily measurable by flow cytometry.^126^, ^127^, ^128^, ^129^ Size‐based subtyping of EVs has well‐founded limitations but, as we shall see later, ApoEVs of nominally sub‐μm sizes may also be categorized as microvesicles (MVs) or exosome‐like EVs and it is currently unclear whether various types of ApoEVs—differing in size and cargo composition—represent a continuum of subcellular particles or whether distinct mechanisms drive their biogenesis (see later Section *ApoEVs and their cargoes* for further discussion). Here, the term ApoBD is used only to describe those bodies arising as a result of apoptotic cell fragmentation. Some reports use the term to refer to the apoptotic cell body itself (for clarity, the term “cell corpse” is used here to denote the latter).

In ultrastructural studies reported in their landmark paper in which they coined the term *apoptosis*, Kerr and colleagues noted frequent encounters with ApoBDs in embryonic, adult and tumor tissues, and provided detailed descriptions of their range of organellar constituents.^25^ A variety of cytoplasmic components are condensed into ApoBDs, including ribosomes, cytoskeletal filaments, endoplasmic reticulum (ER, often dilated), and mitochondria, usually in well‐preserved, morphological states. Sometimes, ApoBDs contain nuclear fragments and condensed chromatin.^25^, ^26^, ^130^, ^131^ They have an approximately spherical or ovoid shape and are smooth in outline, reflecting the loss of microvilli from the surface of their apoptotic cell of origin.^130^ Apoptosis is accompanied (and indeed its mechanism is facilitated) by cell shrinkage caused by loss of water and ions, notably K^+^ and Na^+^,^132^ and this permanent reduction in cell volume, along with the smoothening of the cell surface seems likely to provide much of the additional surface area of plasma membrane required for the delimitation of ApoBDs and perhaps of other ApoEVs too. ApoBD formation and release follows the induction of blebbing at the apoptotic cell surface, initiated by caspase 3‐mediated removal of the C‐terminal inhibitory domain of Rho‐associated coiled‐coil‐containing protein kinase 1 (ROCK1) which, as a result, becomes constitutively activated.^133^, ^134^ Blebbing occurs as a consequence of weaking of the attachments of plasma membrane actin to the underlying cytoskeleton. During apoptosis it is dynamic in nature, many blebs retracting while others are pinched off to release ApoBDs or smaller ApoEVs.^135^ Furthermore, apoptotic cell blebbing displays caspase‐ and ROCK1‐dependent and ‐independent phases.^136^, ^137^, ^138^ Caspase‐mediated activation of ROCK1 is not therefore the defining event in determining fragmentation of the cell into ApoBDs and, as well as dynamic blebbing and ApoBD formation, activated ROCK1 is involved in additional structural changes in apoptotic cells such as shrinkage of the cell body. Once active, ROCK1 phosphorylates myosin light chain kinase (MLCK) which then initiates actomyosin contractility.^133^, ^134^, ^139^, ^140^ Apoptotic bleb formation may also be dependent upon p38MAPK during stress‐induced apoptosis and evidence has been presented that this may be achieved through targeting HSP27, a key regulator of F‐actin polymerization.^141^, ^142^, ^143^ In ApoBD/ApoEV formation, it seems probable that bleb propagation and growth initially follows the same principles as in cytokinesis and cell migration. Thus, detachment of the plasma membrane from the underlying actin cortical cytoskeleton most likely occurs as a consequence of the increase in hydrostatic pressure created by actomyosin contraction which also forces bleb expansion and allows cytosol, together with cytoplasmic organelles, to flow into the bleb as it expands.^144^

In some cell types, including monocytes, fibroblasts, T‐lineage and epithelial cells, blebbing at the apoptotic cell surface is accompanied by the formation of thin surface protrusions that may extend substantially beyond their cell surface origins. Termed apoptopodia and beaded apoptopodia,^128^, ^145^, ^146^, ^147^ these protuberances may represent a specialized step in the budding process that extends the reach of the nascent ApoBDs, promoting their communication with, and uptake by, phagocytes. As yet these structures have only been demonstrated in vitro, mainly in 2D cultures, though recently in a 3D culture system too.^148^ In molecular terms, the control of apoptopodia is not yet well understood, although their production can be upregulated upon inhibition of PANX1. Intriguingly, PANX1 inhibition also promotes production of smaller ApoBDs with greater incidence of nucleoprotein and DNA cargoes.^145^, ^149^ Conversely, in human monocytes efficient production of beaded apoptopodia is dependent upon effector caspase‐mediated cleavage of the transmembrane receptor, plexin B2 (PLEXB2), a regulator of cell migration, cell adhesion and cytoskeletal dynamics.^150^ The significance of the relationships between, firstly, PANX1 channels or PlexB2 receptors and, secondly, apoptopodia‐mediated ApoBD biogenesis/cargo content is not yet fully understood. Further aspects of cargo loading of ApoBDs and other ApoEVs have been reviewed recently.^125^, ^129^, ^151^

Whether or not apoptopodia are involved, the purpose of fragmentation of apoptotic cells through production of ApoBDs remains unclear. It is generally assumed that production of ApoBDs, especially by larger cell types, promotes their capacity to be efficiently phagocytosed. It is certainly the case that small cells such as thymocytes often show no fragmentation as they undergo apoptosis.^130^ Macrophages, dendritic cells and a vast array of so‐called non‐professional phagocytes—which act as effective efferocytes of their apoptotic neighbors include epithelial and endothelial cells, fibroblasts, hepatocytes, satellite, mesangial, Sertoli and Paneth cells as well as various tumor cell types—are capable of engulfing apoptotic cells^25^, ^152^, ^153^, ^154^, ^155^; some efferocytes can engulf whole corpses^156^ even when only minimal morphological apoptotic features are apparent.^157^ It seems reasonable to expect, however, that ApoBD production—disassembly of the dying cell into “bite‐sized” pieces that can be readily engulfed—could be especially important for improving the efficiency of uptake and degradation by the non‐specialized (i.e., non‐macrophage) efferocytes that so frequently engulf their apoptotic neighbors. To some extent this appears to be the case. Effector caspase‐mediated PLEXB2 cleavage and loading into ApoBDs is associated with their improved uptake by non‐specialized efferocytes, basal alveolar epithelial cells in vitro (as well as by alveolar macrophages in vivo).^150^ Furthermore, ROCK1 activation promotes the production of ApoBDs and enhances their clearance by non‐professional phagocytes such as monoblastic leukemia cells, fibroblasts and dendritic cells.^158^, ^159^ ROCK1 seems especially important for ensuring effective clearance in the face of substantive, relatively synchronous, tissue‐wide apoptosis.^140^ It is also probable that fragmentation into ApoBDs facilitates the release of apoptotic cells from mechanical constraints of their surrounding cells and tissue matrix components. Where efferocytosis is undertaken by stationary phagocytes reaching via dendrite‐like processes rather than by whole cell body migration (termed a “stand hunting” strategy^160^) and exemplified by spatially constrained macrophages in the fly,^161^ zebrafish microglia^162^ and mammalian tingible body macrophages (TBMs),^160^, ^163^ fragmentation may be critical for effective clearance by professional phagocytes: in the fly, macrophages can switch, using different actin regulators either to engulf whole apoptotic cells in spacious surroundings using lamellipodia or, when spatially constrained, using fine filopodia to remove apoptotic cell fragments; during brain development in zebrafish, microglia require fragmentation of dying neurons, cells of large size with extensive projections; in mammals, motile fragments allow TBMs to clear massive numbers of apoptotic B cells effectively. In the context of the TME, ApoBD production might be expected to allow for spatial extension of cellular responses to apoptosis, including those of TAMs. Moreover, responses of TAMs and other cell types will undoubtedly be dependent upon the luminal cargoes and surface membrane composition of the ApoBDs they encounter. Recent appraisals of the functional properties of ApoBDs, which include immunomodulation, coagulation and AiP have been provided recently.^125^, ^129^, ^151^

#### 3.1.3 The secretome of cells undergoing apoptosis

Multifactorial microenvironmental signals of relevance to the TME are generated during apoptosis, both through direct release to the extracellular milieu of soluble factors, and via packaging in relatively small (submicron‐sized) ApoEVs. The latter may simply represent the smaller end of the ApoBD spectrum and/or may be generated through MV and exosome pathways. Evidence accrued over several years demonstrates that apoptosis has the capacity to trigger release of a variety of factors which have implications for the cellularity of the TME, for tumor cell growth and survival, for anti‐tumor immunity and, ultimately, for dissemination of malignant disease (reviewed in^164^, ^165^). Although dead (necrotic) cells can passively release a panoply of factors into the extracellular environs, initiation of factor release specifically prior to loss of membrane integrity provides apoptotic cells with an important communicative tool. Notable examples include factors such as TGF‐β1, IL‐10, and alpha defensins^166^, ^167^, ^168^ that contribute to the acclaimed ability of apoptosis to calm inflammation and to suppress adaptive immunity. IL‐10 also has capacity both to promote efferocytosis and to trigger production of BAFF,^169^ a survival factor with a growing reputation in the pathogenesis of multiple cancer types, not restricted to hematological malignancies.^170^ Prostaglandin E_2_ (PGE_2_), a well‐known regulator of the resolution of inflammation (as well as having context‐dependent pro‐inflammatory properties), has been reported to be produced by apoptotic cells to stimulate cell proliferation in tissue repair and regenerative responses.^171^ Due to its multiple oncogenic features, including extracellular mitogen activity, as well as its capacity to promote cell survival and angiogenesis and to operate as a mediator of resolution of inflammation and as an inhibitor of anti‐tumor immunity, PGE_2_ may play a significant part in the oncogenic activities of apoptosis in cancer tissues. Its activity has been observed in several types of cancers and drugs that inhibit its production such non‐steroidal anti‐inflammatory drugs and aspirin may protect against tumor initiation and evolution.^172^

Human apoptotic cells of diverse lineages are able to temper acute inflammatory responses of neutrophils and eosinophils by the de novo synthesis and release of lactoferrin, which radically blocks granulocyte adhesive and migratory capacities and promotes anti‐inflammatory signaling.^173^, ^174^, ^175^ Chemoattractants that are sensed by macrophages early in the clearance phase of apoptosis are among the best‐studied apoptotic cell‐derived secreted factors. These “find‐me” signals comprise multiple categories of molecules including lipids, lysophosphatidylcholine (LPC)^176^ and sphingosine 1 phosphate (S1P),^177^ nucleotides, ATP and UTP,^178^ and the chemokine CX_3_CL1 (fractalkine).^179^

Several studies have clarified how apoptosis‐triggered secretion pathways are linked mechanistically to the apoptosis program. Thus, LPC is closely coupled to the apoptosis machinery, being released as a consequence of the activation of calcium‐independent phosphatase A2 by caspase 3.^176^ The sequence of events leading to the secretion of PGE_2_ is thought to be initiated by effector caspase 3‐ and 7‐mediated cleavage and activation of calcium‐independent phospholipase A_2_ with resultant production of arachidonic acid which is converted to PGH_2_ by cyclooxygenases 1 and 2. Downstream, PGH_2_ is converted into PGE_2_ by PGE_2_ synthase.^171^ ATP and UTP release from apoptotic cells is also linked to a defined effector caspase‐mediated mechanism: the opening of PANX1 channels. Thus, effector caspases 3 and 7 can cleave PANX1 to generate a constitutively open channel configuration that permits release of ATP and UTP (among other relatively small molecules) from the cytosol to the extracellular space.^122^ This mechanism selectively permeabilizes apoptotic cells allowing movement of many molecules of up to approximately 1 kDa between the intracellular and extracellular milieux. In addition to macrophage chemoattractants, of particular interest to the present perspective are the many metabolites that exit from apoptotic cells through PANX1 channels, some of which have been shown to modulate anti‐inflammatory, tissue repair and pro‐survival pathways in phagocytes,^123^ raising the possibility that metabolite transfer to the TME from apoptotic cells could have important consequences for tumor evolution. Intriguingly, apoptotic cells sustain certain metabolic activities, not least the polyamine pathway, the latter study noting that spermidine was substantially secreted from apoptotic cells of various lineages responding to various apoptosis triggers and establishing its release during apoptosis as the first physiological process known to produce extracellular spermidine.^123^ Since spermidine has fundamental and pleiotropic properties and may facilitate or orchestrate either pro‐ or anti‐tumor effects,^180^ the consequences of its release into the TME are worthy of future investigation.

The finding that PGE_2_, produced as a consequence of effector caspases, acts as an extracellular signaling molecule with mitogenic activity for MEFs, stem cell proliferation in wound healing and tissue regeneration^171^ was developed further in model studies of various cancers. Specifically, preclinical studies of mammary and colon carcinoma reported that, following radiotherapy, tumor repopulation was driven by caspase 3‐dependent PGE_2_ production.^74^ Similarly, it was found that, in response to chemotherapy, bladder cancer cells stimulated proliferation of cancer stem cells and tumor repopulation by release of PGE_2_.^181^ Furthermore, caspase 3‐induced apoptosis promoted PGE_2_‐mediated proliferation of melanoma cells^182^ and angiogenesis in glioblastoma following irradiation.^183^ Together, these results are reminiscent of proliferative responses of cell populations that occur consequent to cell death in the fruit fly *Drosophila melanogaster*. These responses were originally termed “compensatory proliferation” (now commonly called apoptosis‐induced proliferation, AiP) and were characterized by apoptotic cells producing mitogens that elicited proliferative signaling pathways in their neighbors.^184^, ^185^, ^186^ Among the factors secreted by apoptotic cells in the fly models investigated (initially wing imaginal disks) are wingless (Wg, orthologue of mammalian WNT) and decapentaplegic (Dpp, orthologue of bone morphogenetic proteins [especially BMP‐2 and ‐4] and TGF‐β), Wg being induced through JNK activation in the dying cells and being necessary for compensatory proliferation.^184^, ^185^, ^186^ Note that JNK and P53 operate in a positive feedback loop both upstream and downstream of the initiator caspase in order to amplify the apoptosis signal.^187^ WNT and BMP/TGF‐β families are functionally conserved from flies to mammals and are known to play significant roles in the regulation of oncogenic pathways.^188^, ^189^, ^190^, ^191^ Thus, the capacity for certain apoptotic cell populations in the fly to elicit compensatory proliferation in their neighbors (as well as the capacity of the neighbors to respond) provides a tantalizing cell death‐mediated extracellular signaling mechanism that may contribute to tumorigenesis in mammals. Of particular note is the observation that in different tissue contexts, AiP can be subserved by different mediators and signaling pathways: in *Drosophila*, in contrast to wing disks which are proliferative tissues where mitogen secretion is initiator caspase‐dependent but effector caspase‐independent, in non‐proliferating cells of the developing eye, mitogen production requires effector caspase activity and critically involves the short‐range signaling protein, Hedgehog (Hh).^192^ Intriguingly, in this context Hh signaling stimulates non‐cycling differentiated cells to re‐enter cell cycle^192^ which has implications for dormant cells in cancer to be triggered into proliferation (e.g., in pre‐metastatic lesions or post‐therapy).

A model of therapy‐induced apoptosis having the capacity to promote oncogenesis in mammals is provided by radiation‐induced thymomas in mice.^47^ In this model, repetition of 1.5 Gy ɣ‐irradiation elicits double‐strand DNA breaks generating oncogenic mutations that drive thymic lymphomagenesis. Whereas deficiency in the apoptosis‐inducing P53‐directed BH3‐only BCL2‐family member, NOXA, caused enhancement of lymphomagenesis in this model, deficiency of its potent cousin PUMA, also a BH3‐only apoptosis‐inducer, surprisingly suppressed tumor development.^47^ It was found that, in PUMA‐deficient animals, re‐establishment of leukocyte apoptosis through glucocorticoid treatment stimulated compensatory proliferation in the quiescent haemopoietic stem cells harboring oncogenic mutations. The mitogenic mechanism remains unclear but strongly supports the view that cycles of AiP have pro‐oncogenic properties in mammals and provides a rationale for therapy‐induced oncogenesis in humans.^47^

Additional evolutionary conservation in repair and regenerative signaling emanating from dying cells is found elsewhere in the animal kingdom. In the cnidarian polyp, *Hydra*, apoptosis has been well established^193^ and, following mid‐gastric bisection, head regeneration is orchestrated through MAPK‐induced apoptosis of interstitial cells at the point of head (but not foot) regeneration. Apoptosis‐triggered secretion of Wnt3, which drove AiP at the site via β‐catenin signaling, was found to be necessary and sufficient for successful head regeneration.^194^, ^195^ Remarkably, this Wnt3‐dependent AiP mechanism is conserved in mammalian hair follicle stem cells.^15^ Caspase activation and apoptosis may also be critical for proper regenerative responses that involve AiP in flatworms, salamanders, frogs, and zebrafish, and although apoptotic cells are commonly observed at the regeneration sites, the extent to which Wnt and/or Hh or other signaling molecules emanate directly from the dying cells remains to be determined (reviewed in^164^). Despite this current lack of molecular detail, and even though molecular mechanisms may vary according to tissue contexts, there seems little doubt that AiP in regenerative responses is evolutionarily conserved, like the apoptosis program itself.

Just as AiP serves to underpin radical replacement of cells in response to wounding or other modes of extensive cell loss, an opposing non‐cell‐autonomous mechanism, apoptosis‐induced apoptosis (AiA) has been described more recently that propagates apoptosis signals in tracts of cells through secretory mechanisms that act over relatively large distances and ensure rapid and synchronous deletion of cell populations. Like AiP, AiA has its origins in *Drosophila* where it was observed that apoptosis initiated in the rear portion of the wing imaginal disk could trigger JNK activation and apoptosis in cells of the anterior aspect of the disc. This phenomenon proved to require production of Eiger (orthologue of mammalian TNF) by the initiated cells.^196^ JNK activation and Eiger production in apoptosis‐initiated cells in the posterior compartment did not inhibit mitogen production by those cells.^196^ In mice, the propagation of apoptosis via this mechanism was observed during the hair follicle cycle of the animals. Notably during catagen, the phase in the hair follicle cycle in which synchronous apoptosis occurs, TNF‐⍺ was found to be produced only by apoptotic cells and antibody‐mediated neutralization of TNF‐⍺ prevented normal propagation of hair follicle apoptosis.^196^ Thus, AiA is an evolutionarily conserved secretory signaling program generated by apoptosis, just like AiP and both processes have strong potential to influence cancer growth kinetics as non‐cell‐autonomous effects of apoptosis (Figure 3).

The secretory activities of apoptotic cells are generally assumed to be served by direct, controlled release mechanisms, such as through effector caspase‐mediated opening of PANX1 channels, as discussed. It is known, however, that apoptosis is associated with increased secretion of ApoEVs and so it is eminently feasible that certain components of the apoptotic cell secretome are released as ApoEV cargo. Indeed, CX_3_CL1/fractalkine provides an illustrative example of a biologically active secreted factor that is released from apoptotic cells, at least in part in association with ApoEVs.^179^ Even when release of molecules occurs through specialized membrane channels such as PANX1, it remains conceivable that such channel activity could occur at the membranes of ApoBDs or other, smaller ApoEVs as well as at the surface of the apoptotic cell body.

##### ApoEVs and their cargoes

Research in the field of EVs has been nothing less than a “growth industry” over recent years and the advancements in knowledge of EV biology as it relates to our understanding of the pathogenesis, detection and treatment of cancer have been substantial. EVs are generally accepted as being lipid bilayer membrane‐encapsulated subcellular shuttling devices produced through a variety of pathways in different sizes (from several nanometers to a few micrometers) by all cell types studied and carrying a wide spectrum of biologically active cargoes, both associated with their membranes and borne in their lumina. Depending mainly on their modes of biogenesis, EVs from healthy cells are roughly spherical or ovoid in shape and comprise two main categories, exosomes and microvesicles (MVs; also known as ectosomes).^197^ Exosomes are commonly found in the size range 30–150 nm and represent the intraluminal vesicles of multivesicular endosomes, while MVs bud directly from the plasma membrane and commonly range from 50 nm to 1 μm in approximate diameter, although may reach >5 μm. Detailed consideration of the history, biogenesis, structure, and function of healthy cell‐derived exosomes and MVs is beyond the scope of the present perspective and the reader is referred elsewhere to excellent reviews on these subjects.^197^, ^198^, ^199^, ^200^, ^201^, ^202^, ^203^, ^204^, ^205^, ^206^ A recent detailed review of the molecular cell biology of ApoEVs is also available.^151^ Although it is well appreciated that EV production is generally increased as a consequence of apoptosis, knowledge of the biological and clinical aspects of ApoEVs lags behind the EV field in general which has been largely focused on EVs derived from healthy cells.

We have already considered one subclass of ApoEV, the relatively large ApoBDs, which are produced as a means of organizing the dismantling of apoptotic cells. Here, we will continue with an overview of smaller ApoEVs (up to 1 μm in size, henceforth referred to as “small ApoEVs”), which will be considered part of the secretome of the apoptotic cell. It is to be expected of course that the arbitrary categorization of ApoEVs by size in this way will fail to define different types of ApoEVs satisfactorily. Here, it is used to facilitate differentiation between (i) fragmentation of apoptotic cells and (ii) secretion of ApoEVs for signaling purposes, representing the main functions of ApoBDs and small ApoEVs, respectively. In reality, these properties of ApoEVs may overlap to a greater or lesser extent. Almost nothing is known about the biogenesis of ApoEVs, apart from those aspects already discussed in relation to ApoBD production, and it is generally assumed that most ApoEVs are generated via bona fide MV biogenesis mechanisms involving direct outward budding of the plasma membrane from the cell surface. However, exosome‐like small ApoEVs have also been reported (see for example^207^). There is no doubt that a fundamental and detailed appraisal of the molecular mechanisms of ApoEV biogenesis and cargo loading, especially of small ApoEVs, is required.

One of the most challenging aspects of EV research is the heterogeneous nature of EV populations, whether from healthy or apoptotic cells. In the case of ApoEVs, sources of heterogeneity relate to the stress signals that elicit apoptosis—which may themselves cause modulation of EV production—as well as to the EV‐inducing effects of the apoptosis program proper and to the variety of biogenesis pathways that may be active, both MV‐like and exosome‐like (reviewed in^151^). ApoEVs contain a diverse array of biologically active cargoes that include organelles and organelle fragments, nucleic acids and proteins, undoubtedly among other, as yet unidentified constituents. They also display in their delimiting membranes features of the plasma membranes of their apoptotic cells of origin (see Section 3.1.3). Thus ApoEVs are commonly (though not universally) reported to display high levels of surface‐exposed PtdSer.^125^ It is noteworthy that this is also a common feature of EVs from healthy cells and, although PtdSer exposure has frequently been used to characterize EVs as arising from apoptotic cells,^151^ this approach clearly lacks specificity for ApoEVs. Indeed, although detailed analyses of the cargoes of ApoEV populations have been undertaken, the most compelling generic diagnostic features of ApoEVs relate to the fragmentation of the nucleus and the characteristic cleavage patterns of genomic DNA in EVs that carry these cargoes.^208^, ^209^ Investigations of these markers have mainly focused on ApoBDs to date and future work would therefore benefit from broader studies of ApoEVs of all sizes and origins. More detailed investigations may also identify additional cargoes or surface markers of ApoEVs specific to certain contexts, such as particular cancer types or disease stages. For example, ApoEVs are known to display not only surface markers that are characteristic of their cell lineages of origin, but also altered surface features such as C1q‐binding sites and other apoptotic cell‐associated molecular patterns^151^ that may be of diagnostic value and also important for interactions with recipient cells such as efferocytes which are known to deploy numerous pattern recognition and scavenger receptors in order to engage with apoptotic cells (Section 3.2.1). Obviously, physical size will constrain both luminal cargoes and surface composition of ApoEVs.^210^

From the standpoint of the TME, it seems reasonable to speculate that secretion of biologically active molecules in the format of ApoEVs ensures protection of labile factors from degradation, thus promoting the longevity of the apoptotic cell's signaling capacity and extending its spatial properties. In addition, molecular clustering within or on the surface of ApoEVs provides opportunities for signaling via complexes of molecules—perhaps to promote binding avidity—or providing appropriate structural topology such as surface curvature which may be required for receptor ligation on responding cells. Considering their known constitution and cargoes, ApoEVs possess a range of attributes of potential functional significance for the TME (although little direct evidence is yet available). Their membranes, through exposure of PtdSer and Annexin A1 for example, may be expected to have anti‐inflammatory properties.^100^, ^211^ Some surface components, namely CX_3_CL1/fractalkine and ICAM‐3, are known chemoattractants for macrophages.^179^, ^212^ Other ApoEV constituents including DNA, RNA, internucleosomal histones, ER fragments, ribosomes, and aggregates of ribonucleoproteins are common autoantigens^135^, ^213^, ^214^, ^215^, ^216^, ^217^, ^218^, ^219^, ^220^ and can elicit inflammatory and autoimmune responses in certain contexts, notoriously under conditions of impaired efferocytosis.^154^, ^221^, ^222^

Various functions of small ApoEVs have been proposed, including pro‐inflammatory, anti‐inflammatory, immunomodulatory and tissue repair effects, although in many studies the ApoEV populations have not been well defined and little is yet known about modes of interaction with recipient cells (reviewed in^151^). ApoEVs may also be active in the horizontal transfer of double stranded DNA^223^ from apoptotic tumor cells to either neighboring tumor cells or stromal cells as has been described for exosome‐like EVs in cancer.^224^ In the cancer context, a recent study of small ApoEVs in glioblastoma is compelling.^225^ As noted from Table 1, in glioblastoma, substantial apoptosis occurs next to necrotic cores^34^ and it has been estimated that as much as 70% of the cells are apoptotic.^225^ Intercellular signaling mediated by small ApoEVs from aggressive glioblastoma clones was found to impart aggressive features of enhanced proliferation, migration, and anti‐cancer drug resistance on more benign recipient clones. The underlying molecular mechanism involved ApoEV‐mediated transfer of spliceosome components such as RBM11 which promoted the expression in recipient cells of isoforms of MDM4 and CCND1 (cyclin D1) having increased oncogenic activities.^225^ It is noteworthy that, although the mode of cell death was not defined in the investigation, dying pancreatic cancer cells following irradiation released small EVs that promoted recovery of residual tumor repopulating cells through transfer of miR‐194‐5p and subsequent PGE_2_ secretion which stimulated tumor cell proliferation.^226^

### 3.2 Communication of apoptotic cells with TAMs

Macrophages are residents of all our tissues. Based on their exquisite microenvironmental sensitivity, macrophages' profound importance lies not only in their capacity to respond immunologically to infection or damage, but also fundamentally in the regulation of tissue homeostasis.^227^ In cancers too, macrophages subserve critical inflammatory and homeostatic (albeit aberrant) functions and TAMs have become especially renowned for their pro‐oncogenic supportive roles, even though they also have proven anti‐cancer potential and may even help in anti‐tumor immune surveillance. TAMs are widely accepted as major players (and are often also major constituents) among the cells of the TME. Overwhelming evidence from wide‐ranging cancer types including lymphoma, glioblastoma, breast, melanoma, colorectal, bladder, ovarian, and other cancers indicates that TAMs are associated with poor prognosis. This association is linked to their capacity to be critically influenced by primary and metastatic TMEs to adopt reparatory and regenerative activation states. Here, discussion focuses specifically on the roles and potential roles of apoptotic cells in driving such activation and the reader is referred to several excellent reviews for more detailed, broader aspects of macrophage and TAM biology.^228^, ^229^, ^230^, ^231^, ^232^, ^233^, ^234^, ^235^

#### 3.2.1 Macrophage efferocytosis and its implications for the TME: The “3Rs” of apoptosis

Efferocytosis is essential to the homeostatic value of apoptosis (reviewed by^8^, ^98^, ^154^, ^236^, ^237^, ^238^ and, as alluded to already, many of the changes at the apoptotic cell surface have proven utility in coupling with receptors on macrophages (as well as on other, “non‐professional” efferocytes) in *recognition* of the dying cell. This leads to *response* signaling by the macrophage which in turn leads—among other responses—to *removal* by phagocytosis and subsequent digestion through defined pathways. This recognition/response/removal scenario (the “3Rs” of apoptosis^28^, ^239^) lies at the heart of tissue homeostasis that is controlled by apoptosis (which is fundamental to basal tissue turnover) and appears tailored to heightened requirements when apoptosis increases, such as in the resolution of acute inflammation. Typically, macrophages responding to apoptosis do so non‐phlogistically: either without affecting inflammation, or by producing anti‐inflammatory mediators such as TGF‐β1, IL‐10 and, in this context, PGE_2_, as well as down‐regulating pro‐inflammatory mediators such as TNF‐⍺, IL‐1β, IL‐6 and IL‐12.^230^, ^240^ Indeed, apoptotic cells drive anti‐inflammatory, pro‐repair (M2‐like) phenotypes in macrophages and can dominantly inhibit the classically activated, pro‐inflammatory and anti‐tumor (M1) activation state.^241^, ^242^, ^243^ In certain circumstances, for example through translocation of CRT to the apoptotic cell surface or, as indicated, through production of specific types of ApoEVs, the macrophage response to apoptosis can be pro‐inflammatory, and both pro‐ and anti‐inflammatory response categories have implications for the TME.

##### Recognition

Macrophage recognition of apoptotic cells and bodies begins with sensing the initiation of apoptosis. Appropriately described as the “smell phase,” macrophages respond to secreted chemoattractant factors from apoptotic cells including LPC, ATP/UTP, CX_3_CL1/fractalkine and S1P, all of which can activate mononuclear phagocytes to migrate directionally through G protein‐coupled receptors.^244^ Chemoattractants are also able to prepare macrophages for efficient corpse clearance and to help induce a non‐phlogistic environment. ATP, for example, can upregulate the efferocytic integrin, a_v_β_3_ on macrophages^245^ and extracellular metabolism to AMP and adenosine can orchestrate anti‐inflammatory and immunosuppressive responses.^246^, ^247^ Fractalkine can promote production of MFG‐E8 and efferocytosis by macrophages and reduce tissue injury in a sepsis model.^248^, ^249^ Fractalkine has established anti‐inflammatory activities, such as in suppressing TNF‐⍺ secretion and neurotoxicity of microglia stimulated with LPS.^250^ Of relevance to the TME, CX_3_CR1 signaling promotes proangiogenic TAM accumulation in aggressive malignant disease^251^ and S1P also appears to regulate pro‐tumor activation and accumulation of TAMs. Of further interest is the stimulation of production of the pro‐resolvin PGE_2_ following S1P activation of macrophages.^230^ PGE_2_ can also upregulate the apoptotic‐cell tethering receptor CD14 by macrophages.^252^ The potency of these migratory stimuli relative to other chemoattractants sensed by macrophages in mammals is unknown, but it is noteworthy that in *Drosophila*, hemocyte (fly macrophage) migration in response to apoptotic events in the embryo is dominant.^253^ Given the dominance of apoptosis in driving mammalian macrophage activation, it is tempting to speculate that dominant migratory responses to apoptosis will also be evolutionarily conserved. As we have seen, tissue resident macrophages directionally extend dendrite‐like processes to engage with apoptotic cells rather than undergoing cell‐body migration. This “stand hunting” capacity has been well studied in the context of microglial cells, the macrophages of the central nervous system and of the TBMs found in the germinal centers (GCs) of lymphoid tissues, although it is not yet clear how their processes “smell” apoptosis. It is of course conceivable that a subset of the very same sensing and membrane migration processes that are involved in chemotaxis also apply to stand hunting.

The second stage of recognition is the establishment of avid binding between the apoptotic cell and the macrophage. This has been termed the “taste phase” or establishment of a “phagocytic synapse” and involves engagement (either directly or indirectly, as already discussed) of the new features of the apoptotic cell surface with macrophage receptors. Some of the receptors are able, not only to bind the apoptotic cell, but also to signal to the phagocyte, while others do not have inherent capacity to activate phagocyte signaling alone. This combination of receptor‐ligand interactions that initiates corpse engulfment has aptly been termed “tether and tickle” and involves a blend of different pathways—“bind‐me,” “eat‐me” and loss of “do‐not‐eat‐me” signals—that cooperate to activate the phagocytic response, and possibly other (notably anti‐inflammatory) responses too. The extent to which the constitution of the phagocytic synapse differs in different tissue, contexts including TMEs, is unclear; the array of deployable receptors however, provides ample scope for tuning of the recognition phase of efferocytosis for different phagocytes in preparation for various downstream responses, and possibly also for discriminatory responses to different temporal phases of the apoptosis program or to different products of the apoptotic cell including ApoBDs and other ApoEVs, in addition to the corpse itself.

##### Response

Knowledge of the post‐recognition responses of macrophages is, unsurprisingly, dominated by the corpse engulfment signaling which is ultimately mediated by the Rho GTPase, Rac1 and its downstream effectors that regulate cytoskeletal rearrangement and phagocytic cup formation.^154^ During apoptosis in the nematode worm, *C. elegans*, two pathways converge on the Rac1 orthologue, CED‐10 (cell death defective gene‐10) and molecular players are conserved in mammals^254^: the first pathway involves CED‐1, ‐6, and ‐7 while the second sequence upstream of CED‐10 comprises CED‐2, ‐5, and ‐12. In the first pathway, the mammalian orthologues of CED‐1 are the receptors CD91/LRP1 or MEGF10; of CED‐2, the adaptor GULP1 which operates downstream of CD91/LRP1; and of CED‐7, the ATP‐binding cassette transporters ABCA1 and ABCA7. The precise roles of the latter transporters in mammalian corpse engulfment remain contentious, although ABCA1 is involved both in the release of LPC from apoptotic cells^255^ and in the recycling of cholesterol from phagocytes.^256^ In the second pathway, the mammalian orthologue of CED‐2 is CrkII; of CED‐7, ELMO1; and of CED‐12, DOCK180. The role of CrkII is not entirely clear, but ELMO1 and DOCK180 act in complex as a guanine nucleotide exchange factor (GEF) for Rac1 activation (reviewed by^240^, ^257^). Acting upstream of ELMO1/DOCK180, CD91/LRP1 exemplifies receptors outlined earlier, PtdSer‐dependent and PtdSer‐independent, that undertake not only recognition of apoptotic cells, but also post‐recognition engulfment responses. The direct PtdSer receptor, BAI1 also activates Rac1 and consequent engulfment via ELMO1/DOCK180.

Blockade of apoptotic cell clearance is most renowned in mammals for its autoimmune consequences. As yet, little is known about its implications for cancer, although there is little doubt that the TYRO3‐AXL‐MER family of receptor tyrosine kinases which mediate both apoptotic‐cell engulfment and anti‐inflammatory signaling, can influence malignant disease pathogenesis both positively and negatively. Mechanistic details are complex and pleiotropic, however, because these receptors, especially AXL and MER are known to be active in cancer cells, in addition to their activities in macrophages and other cell types in the TME.^258^ Nevertheless, clinical trials of several AXL and MER inhibitors are in progress for treatment of a spectrum of tumor types including glioblastoma, breast and lung cancers, and acute leukemia.^98^ Specific effects of MER‐mediated efferocytosis have been established in preclinical cancer models, an excellent illustrative example being that of metastatic postpartum breast cancer. Clearance of dying tumor cells was found to be MER‐dependent and associated with the production of reparatory cytokines such as TGF‐β1, IL‐4, IL‐10, and IL‐13. MER‐deficiency or MER‐ or TGF‐β‐blockade inhibited M2‐like activation of TAMs (though not TAM numbers) and reduced metastases.^259^ Detailed additional evidence linking AXL‐ and MER‐dependent efferocytosis with the generation of pro‐oncogenic, anti‐inflammatory milieux has been reviewed recently.^98^, ^260^

The anti‐inflammatory and M2‐like phenotype activated in macrophages responding to apoptotic cells is reminiscent of that of wound‐healing macrophages and aligns with the M2‐like, pro‐tumor activation state of TAMs in general (discussed further in Section 3.2.2 below). During the resolution phase of inflammation generated by skin wounding in mice, clearance of apoptotic inflammatory cells (especially neutrophils) drives the M2‐like phenotype of the wound‐healing macrophages, including expression of CD206 and arginase I, two cardinal features of the reparatory macrophage phenotype.^261^, ^262^ This accords with the previously discussed principle of apoptosis orchestrating TME features that resemble healing wounds. A novel anti‐inflammatory regulatory pathway triggered by efferocytosis was recently reported to be mediated by the chloride transporter SLC12A2 and its associated kinases involved in chloride sensing. This sensor may act as a molecular switch in efferocytes, since its downregulation or functional inhibition can flip the phagocyte phenotype from anti‐inflammatory to pro‐inflammatory. SLC12A2 chloride sensing also moderates efferocytosis, preventing “over‐eating” by efferocytes.^263^

Additional relevant responses include the production of VEGF by macrophages that engulf apoptotic cells,^264^ which, as well as its prominence in angiogenesis, has the capacity to control many physiological processes including promotion of endothelial cell survival, vascular leakage and migration of myeloid cells,^265^ all of which have strong relevance to cancer biology. As discussed later, efferocytes have the potential to produce a range of factors that promote cell survival, proliferation and migration/invasion all of which may have important implications for the hijacked homeostatic functions of the TME. Of note is the possibility that TAMs may be influenced by contact with apoptotic cells in the TME, regardless of whether this is followed by phagocytosis. In this way, the contacting membranes of apoptotic cell bodies, ApoBDs and other ApoEVs may operate as potent signaling entities through macrophage receptor engagement alone and this may be extended to cells other than macrophages that display appropriate receptors.

##### Removal

The final phase in the clearance process is phagolysosomal maturation, acidification, and hydrolytic degradation, the molecular cell biology of which has been reviewed recently.^154^ It has long been assumed that, given the voracious efficiency with which they are able to consume dying cells, macrophages have in place robust salvage and recycling mechanisms in order to deal effectively with such challenging metabolic burdens. Indeed, the capacity for recycling the constituents of dying cells would be expected to be a particular feature of proliferating tissues—including cancers—in which cell death is a common occurrence. This recycling property of efferocytosis is well illustrated by apoptotic cell‐derived cholesterol which has been shown to efflux effectively through the ABCA1 transporter of macrophages. The pathway is activated by BAI1 following ligation by PtdSer and subsequent Rac1 activation via ELMO1.^256^, ^266^ A complementary pathway of cholesterol efflux is provided by the liver X receptor (LXR) which also operates to promote MER expression and anti‐inflammatory signaling.^267^

Other modes of coupling between metabolic recycling and anti‐inflammatory, pro‐resolution signaling pathways of efferocytosis have been reported. For example, a speedy degradative response to engulfed apoptotic cells is associated with phagosomes that have been modified by microtubule‐associated protein 1A/1B light chain 3 (LC3). LC3‐associated phagocytosis (known as LAP) engenders anti‐inflammatory responses, including IL‐10 production, and immunological silence. LAP may operate through its ability to drive M2‐like macrophage activation, not least by metabolic reprogramming (see^268^ for detailed review). Reparatory M2‐like polarization and transcriptional activation of *Il10* have been reported to arise in efferocytosing macrophages from recycling of fatty acids. This pathway is dependent upon NAD^+^ and SIRTUIN1, which activates the *Il10* transcription factor, PBX1.^269^ Furthermore, synthesis of PGE_2_, an important resolvin produced as a consequence of efferocytosis, is induced following engulfment via the scavenger receptor CD36, of multiple apoptotic cells. PGE_2_ subsequently activates TGF‐β1 and repair programming. Prolonged activation of the pathway is achieved through the action of recycled, apoptotic cell‐derived methionine which, after conversion to S‐adenosylmethionine, acts as a substrate for DNA methylation by DNA methyl transferase 3A resulting in downregulation of the ERK1/2 phosphatase DUSP4 which permits sustained induction of ERK‐COX2‐PGE_2_‐TGF‐β1 reparatory signaling.^270^

Apoptotic cells have been reported to induce mitochondrial fission by dynamin‐related protein 1 in phagocytosing macrophages, which was found to be essential for effective clearance of a high apoptotic cell burden.^271^ More recently, recycling of arginine and ornithine from apoptotic cells in phagocytes was also found to be important for continuing efferocytosis. The mechanism involves metabolism of arginine and ornithine by arginase 1 and ornithine decarboxylase, respectively, resulting in putrescine‐driven Rac1 activation via the GEF, Dbl.^272^ In addition, expansion of the efferocytosing macrophage population can be induced by recycling of apoptotic cell‐derived DNA. Thus, phagolysosomal DNA hydrolysis by DNase2A within the phagocyte triggers a PKcs‐ and mTORC2/Rictor‐dependent pathway that increases Myc expression and drives proliferation of reparatory macrophages. Intriguingly, this pathway was found to be coupled to the engulfment receptor MER.^273^

Together, these results demonstrate that degradation of engulfed apoptotic cells and recycling of their components not only provide for the activation of the reparatory phenotype of efferocytosing macrophages but also ensure sufficiency of efferocytic and tissue repair capacity, both through “tune‐up” of individual macrophages and through expansion of the macrophage pool. Although yet unproven, these characteristics have important implications for best use of metabolic resources in the TME.

#### 3.2.2 Starry‐sky lymphoma: Modeling the apoptotic “niche” of TAMs

The descriptive starry‐sky imagery that depicts, in standard histological sections, pale‐staining TAMs dotted in a “sky” of darkly stained tumor cells, can be observed in hematological malignancies, prototypically BL but also in many others, including diffuse large B‐cell lymphoma, plasmablastic lymphoma, mantle‐cell lymphoma, B‐ and T‐lymphoblastic leukemia/lymphoma and peripheral T‐cell lymphoma^274^; it can also be seen in non‐hematological malignancies such as angiosarcoma.^275^ In BL and other starry‐sky non‐Hodgkin's B‐cell lymphomas, the TAMs, interspersed among the rapidly expanding tumor cells, create a microarchitecture that resembles that of the GC reaction of secondary lymphoid follicles, a normal immune response in which rapid B‐cell expansion is required for production of clones carrying affinity‐matured antibody specificities following hypermutation of immunoglobulin variable‐region genes. In GCs, the starry‐sky macrophages are termed TBMs because they are laden with the darkly stained, pyknotic remnants of apoptotic B cells that have failed the stringent antigen‐dependent selection processes required to save high‐affinity clones. Starry‐sky macrophages in B‐cell lymphomas (SS‐TAMs) similarly reflect the high apoptosis rate of the tumors they inhabit and, like TBMs, are overtly, often heavily, laden with the remains of apoptotic cells. Starry‐sky lymphomas therefore represent an intriguing opportunity to study the biology of TAMs in a TME in which proliferation and apoptosis occur together at high rates and in which TAMs have an obvious, efferocytic relationship with apoptotic tumor cells.

We have used mouse models of starry‐sky lymphoma, including BL, to demonstrate how apoptosis in the tumor‐cell population can influence tumor growth, angiogenesis, SS‐TAM accumulation and activation.^276^ Inhibition of apoptosis using either BCL2 or BCL‐xL promoted expansion of tumor cell populations in vitro but, surprisingly, constrained their growth in vivo. In parallel, TAM accumulation was markedly suppressed under conditions of apoptosis inhibition as was angiogenesis. Furthermore, SS‐TAMs were found to proliferate in situ and transcriptomic profiling of SS‐TAMs isolated by laser capture microdissection showed them to be activated to an M2‐like reparatory state and included upregulation of genes involved in efferocytosis and anti‐inflammatory signaling, including *Lrp1*, *Mertk* (the gene for MER), *Axl*, *Gas6*, *Tgfb1*, and *Lgals3* (the gene for galectin 3).^276^ Of note, *Mertk* ^−/−^ or *Lgals3* ^−/−^ mice were found to be defective in their ability to support starry‐sky lymphoma growth^243^, ^277^ and the gene expression profiles of SS‐TAMs were predicted not only to signal effective uptake and anti‐inflammatory/immunosuppressive responses to apoptotic tumor cells but also potentially to produce trophic, angiogenic, and invasive factors for tumor growth and evolution.^276^

Extending beyond morphology, many of the features of SS‐TAMs reflect those of TBMs, both macrophage types being highly active in efferocytosis of substantial numbers of apoptotic cells, both deploying MER (likely among other receptors) and both proliferate in situ. Recent work also demonstrates that TBMs of GCs are produced by proliferation of resident lymphoid tissue macrophages whose capacity for expansion and for efficient efferocytosis through extension of dendrite‐like projections to pick up ApoBDs by a stand hunting process is driven by apoptosis.^160^, ^163^ It seems reasonable to speculate that SS‐TAMs function similarly. Intriguingly, TBMs, unusually for macrophages, expand independently of CSF‐1^163^ and this has also been reported in other, independent studies of efferocytosis‐induced, MER‐dependent macrophage proliferation in a non‐GC setting.^273^ Also fibroblasts participate in the regulation of tissue macrophage numbers,^278^ although this has not been studied in the specific context either of TAMs or TBMs. It remains to be seen what if any apoptotic cell‐driven attributes of TBMs are provided to the GC niche.^279^ It seems probable that much knowledge could be gained about the apoptosis‐driven contributions of TAMs to the TME by further, formal comparisons of SS‐TAMs and TBMs.

### 3.3 An apoptosis‐centric perspective of the tumor microenvironment

Cancer may be broadly considered as a condition characterized by the acquisition of aberrant tissue homeostasis. Within this framework, apoptosis is highlighted here as a fundamental cellular program, that not only (1) helps determine cell numbers in cancerous tissues through cell‐autonomous deletion, but also (2) contributes to control of cell population sizes through non‐cell‐autonomous effects (Figure 3). While both of these aspects can cause growth suppression on pre‐neoplastic or neoplastic populations (exemplifying apoptosis‐mediated anti‐cancer surveillance or remission), the second principle provides, in effect, negative feedback to cell death at the tissue homeostatic level, sustaining net growth and possibly contributing too to the generation of hardier, more aggressive clones having “winner” status under the rules of cell competition as it pertains to proliferation, apoptosis and efferocytosis.^280^ Because of its significance, firstly as a key supportive cell type in tissue homeostasis^281^ and cancer development and progression, and secondly in efferocytosis, the macrophage very likely plays a critical part in the apoptosis‐centric function of the TME. In addition, many other cells that are major cellular constituents of the TME, including fibroblasts, endothelial cells and others, together with tumor cells themselves, have efferocytic activities and can be regulated by apoptotic cells and their products. Importantly, whether or not they are overtly phagocytic, tumor and stromal cells may have capacity to respond to apoptotic neighbors either through direct contact or through engagement with apoptotic cell‐derived factors delivered in soluble form or via ApoEVs (Figure 4).

It seems likely, therefore, that most if not all cells in the TME are subject to the effects of apoptosis in their neighborhood.^28^, ^165^ It has been reported that, independently of the phagocytic response, apoptosis has the ability to elicit contact‐dependent, phagocytosis‐independent repression of inflammatory signaling in macrophages^282^ and a consistent “innate apoptotic immune signature,” unrelated to phagocytosis, in phagocytes and non‐phagocytes alike upon interaction with apoptotic cells. An emerging principle which has highly significant implications for the aberrant homeostasis of cancer tissues involves the control of metabolite fluxes, both between different intracellular compartments and across the intracellular and extracellular milieux, by the large superfamily of solute carrier (SLC) transporters. Communication with apoptotic cells has profound effects on the biology of SLC transporters in neighboring cells, including professional and non‐professional efferocytes and eliciting modulation of multiple SLCs regulating diverse cellular activities ranging from cell volume control to carbohydrate metabolism and cytoskeletal rearrangement. Contactless sensing of apoptotic cells, for example, was found to be sufficient to trigger activation, by SGK‐1, of the glucose transporter, SLC2a1 increasing its plasma membrane presence. Contact via PtdSer/receptor interactions increased SLC2a1 expression at the transcriptional level and the resultant upregulated glucose uptake and glycolytic activity permitted not only the activation of initial engulfment events, but also supported continued clearance of multiple corpses.^283^ Furthermore, engulfment triggered transcriptional activation of the lactate transporter, SLC16a1, thereby enhancing efflux of lactate from the cell and creating a demonstrably anti‐inflammatory environment characterized by its ability to transcriptionally activate an M2‐like program in macrophages, including upregulation of *Tgfb1*, *Il10*, *Vegfa*, *Mgl1*, and *Mrc1*.^283^ The implications for this SLC transporter axis in the TME are clear: Originating from aerobic or anaerobic glycolysis, lactic acid produced by tumor cells has an established role in polarizing TAMs to an M2‐like anti‐inflammatory, pro‐reparatory state and promoting tumor growth^284^; modulation of the SLC2a1/SLC16A1 axis provides a potential mechanism for apoptotic tumor cells to perform a similar, pro‐oncogenic role (Figure 4).

Intercellular communications circuitry in response to apoptosis is therefore likely to play important roles in cancer tissue programming. Further examples which have potential to contribute to aberrant homeostasis in cancer tissues are noteworthy. IGF‐1 is renowned for its ability to facilitate cancer development through its capacity to stimulate growth and inhibit apoptosis. Macrophages phagocytosing apoptotic cells have been shown to produce IGF‐1 and also to secrete MVs, both of which affect neighboring epithelial cells. The latter non‐professional efferocytes were stimulated by IGF‐1 to reduce their phagocytosis of apoptotic cells in favor of internalizing MVs which suppressed their inflammatory responses.^285^ Cross‐talk between injured epithelia and macrophages has also been found to establish a circuit that mediates epithelial regeneration. Thus, induction of apoptosis in airway epithelial cells triggered caspase‐dependent PANX1 opening and consequent release of ATP and other factors that engender a form of epithelial AiP through induction of amphiregulin, an epithelial mitogen, by macrophages responding to the epithelial apoptosis.^286^ PtdSer‐independent efferocytosis of apoptotic intestinal epithelial cells via the C‐type lectin receptor, SLECTin appears to stimulate similar regenerative activity in gut epithelia,^120^ although the post‐efferocytic effector mechanism has not yet been elucidated. These examples serve to illustrate the complexities of the intercellular communications networks that derive from apoptosis and suggest that context‐dependent circuits of similar intricacy will also prove to operate in cancer tissues.

Sensitivity of tumor cells to activation of their apoptosis program, which is orchestrated by both cell‐intrinsic and cell‐extrinsic factors, is a crucial determinant in the tumor growth equation. The long‐standing model of apoptosis thresholding has useful attributes, not least that cell fate is unlikely to be determined by one specific signal change.^45^ In this model, a cell's ability either to keep suppressed its latent apoptosis program or alternatively to activate it, is determined by the activity balance between pro‐survival (BCL2, BCLxL, MCL1, XIAP, AKT, etc) and pro‐apoptotic (Caspases, BAX, BAK, BIM, PUMA, NOXA, FAS, etc) signal mediators. Sensitivity to apoptosis thresholding may be maintained from pre‐neoplasia to established malignancy and even aggressive stages of disease. Intriguingly, apoptosis thresholding increases in normal tissues with age as a consequence of downregulation of components of the apoptosis machinery. In young mice and humans, thresholding is reduced and expression of pro‐apoptosis program proteins is driven by c‐MYC.^287^ This mechanism accords with the well‐established coupling of developmental cell growth to apoptosis and may underlie the strong association between apoptosis and proliferation in tumors as we have seen.

Related to this, failed apoptosis and nonapoptotic functions of the apoptosis machinery have implications for tumor emergence and progression (reviewed in^288^, ^289^, ^290^). Non‐canonical functions of caspases, for example, may be important in driving proliferation^291^ and EV biogenesis and activities.^292^ In *Drosophila*, it was found that translocation of the initiator caspase, Dronc to the plasma membrane by the unconventional myosin, Myo1D can activate AiP‐like proliferation, independently of apoptosis through activation of the fly NADPH oxidase, Duox which generates reactive oxygen species.^293^ Surprisingly, sublethal caspase 3 activation can drive genomic instability of c‐Myc and resultant oncogenic transformation in mammalian cells.^294^ Failure of completion of the apoptosis program can also occur in cells in which only a minority of mitochondria undergo critical apoptosis‐initiating events downstream of MOMP. Intriguingly, cells displaying such features (“minority MOMP”) undertake sub‐apoptotic caspase activation that permits survival, genomic instability, and oncogenesis.^295^ Furthermore, cells undergoing apoptotic stress following BH3 mimetic drug treatment can release the trophic factor, FGF2 regardless of completion of their apoptosis program. The secreted FGF2 promotes survival of neighboring cells through MEK/ERK‐induced upregulation of BCL2 or MCL1 and leads to anti‐cancer drug resistance.^296^

A consensus view of the potential pathways through which the apoptosis program—whether induced pathophysiologically or therapeutically—can influence the TME is summarized in Figure 4. These pathways are expected to vary in different cancer types and at different stages of disease progression.

## 4 CONCLUSIONS AND FUTURE PERSPECTIVES

Accumulated knowledge of the molecular detail of the apoptosis program, regulation of its machinery, and its communicative roles in inflammation and tissue homeostasis provide a firm foundation for better understanding of the contributions of apoptosis to malignant diseases. Of course, as a cell death modality, apoptosis does not occur in isolation, even though in growing tumors it is likely to be the most common of the regulated cell death programs. By way of illustration, it has been suggested that necrosis, a frequently encountered mode of accidental, passive and unregulated cell death, has oncogenic properties, probably derived from its ability to stimulate tissue repair^297^ and mediated through its pro‐inflammatory effects becoming chronic.^298^ Like apoptosis, necrosis can also generate extracellular signals that mediate compensatory proliferation at least in certain contexts. Necrotic hepatocytes, for example, have been reported to release IL‐1⍺ which promotes chemical carcinogen (DEN)‐induced compensatory proliferation that drives HCC.^299^ Moreover, different modes of regulated cell death have proven properties in determining the direction of cancer evolution. Thus, in transposon models of primary liver cancer in mice, it has been shown that a regulated form of necrosis, necroptosis, drives transformed hepatocytes toward cholangiocarcinoma, while an apoptotic environment selects for development of HCC.^300^ The different liver cancer models of HCC, with necrosis promoting chemical carcinogenesis in the former study^299^ and apoptosis facilitating the latter^300^ also suggest that different routes to development of the same cancer type may be directed by different modes of cell death. In addition, “cooperation” between different subroutines of regulated cell death may occur to promote tumor growth, a recent report suggesting that pro‐inflammatory efferocytosis of apoptotic cells, caspase 1‐induced, gasdermin D‐independent, NLRP3‐dependent inflammasome activation and resultant IL‐1β production has tumor‐promoting properties which may be important in inflammation‐driven tumor progression in head and neck squamous cell carcinomas.^301^ Other homeostatic programs affecting cell survival and population dynamics such as autophagy^302^ and senescence^303^ will undoubtedly also play key contributory roles in oncogenesis and progression of malignant diseases in the complex circuitry of cancer tissues alongside apoptosis and other cell death modalities, including regulated, caspase‐independent cell death, which can be mediated in certain contexts by EVs.^304^

Understanding the integration of the multitude of cell death and environmental signals that determine specific efferocyte responses is hugely challenging (reviewed in^305^) and one of the most intriguing questions in the apoptosis field is how (and why) so many receptors and opsonins are deployed in efferocytosis. Among other possibilities, this may relate to redundancy, cell and tissue context, and functional variation. Certainly, some receptors such as CD14^29^, ^116^ appear to function only in tethering of apoptotic cells (especially lymphocytes) to phagocytes, whereas others, for example MER are active signal transduction receptors, triggering both engulfment and anti‐inflammatory signaling pathways in responsive efferocytes. Furthermore, cell context can change the signaling response that follows apoptotic‐cell recognition in the “taste” phase. This is illustrated by MER's capacity to activate signal transduction pathways either for efferocytosis or for proliferation which are dependent upon different as well as overlapping cytoplasmic domains of MER. Furthermore, the ability of apoptotic cells to stimulate proliferation through MER was found to require TIM4.^306^ An additional possibility is that the apparent variety of efferocytic pathways provides opportunities for diversification of signaling responses to ApoEVs, as well as apoptotic cells.

To conclude, there seems little doubt that apoptosis holds much promise as a significant, evolutionarily conserved regulator of “hijacked homeostasis” in tumor biology, but despite the signaling qualities of apoptotic cells becoming ever clearer, many of the underlying oncogenic principles remain largely theoretical. Most of the important questions remain unanswered, not least the following: (1) How do the roles of apoptosis vary in different cancer types, in different stages of an individual cancer, and in different regions of a primary or metastatic lesion? For example, how does the apoptosis‐driven phenotype of TAMs or the secretome of apoptotic tumor cells vary in these scenarios? (2) Focusing on the macrophage as an important regulator of tissue homeostasis, what are the phenotypic differences between efferocytic normal tissue resident macrophages and the efferocytic TAMs in their counterpart malignant tissues? (3) Can cancer therapies be improved by drug‐targeting apoptosis‐induced pro‐tumor responses in combination with conventional apoptosis‐inducing radiation or chemo‐therapies? (4) Can products of apoptotic cancer cells or their responding cells provide useful biomarkers for early cancer diagnosis or detection of minimal residual disease? The scene is set and the technologies, such as spatial multiomics,^307^ along with ground‐breaking constructs to detect sequential stages of apoptosis from initiation to degradation (e.g.,^30^) are ready to be applied to help answer these and related questions in the near future.

## CONFLICT OF INTEREST STATEMENT

The author declares no conflict of interest.

## Data Availability

Data sharing is not applicable to this article as no datasets were generated or analyzed during the current study.
